# Zolpidem restores sleep and decreases amyloid in a mouse model

**DOI:** 10.1002/alz.71175

**Published:** 2026-03-11

**Authors:** Lu Yu, Shinya Yokomizo, Tri H. Doan, Qiuchen Zhao, Akshatha Ganne, Meenakshisundaram Balasubramaniam, Ksenia V. Kastanenka

**Affiliations:** ^1^ Department of Neurology MassGeneral Institute of Neurodegenerative Diseases Mass General Brigham and Harvard Medical School Charlestown Massachusetts USA; ^2^ Department of Neurology The Affiliated Hospital of Xuzhou Medical University Xuzhou Medical University Xuzhou China; ^3^ Department of Geriatrics University of Arkansas for Medical Sciences Little Rock Arkansas USA

**Keywords:** Alzheimer's disease, amyloid plaques, APP/PS1 mice, calcium homeostasis, GABA_A_ receptor, memory consolidation, neurodegeneration, NREM sleep, sleep, slow oscillation, zolpidem

## Abstract

**INTRODUCTION:**

Deficits in non–rapid eye movement (NREM) sleep facilitate Alzheimer's disease (AD) progression. Enhancing gamma‐aminobutyric acid‐ergic (GABAergic) signaling can restore sleep. Unbiased computational analysis identified zolpidem as high‐affinity GABA receptor modulator facilitating chloride transport that could slow AD.

**METHODS:**

Zolpidem's effects on sleep and Alzheimer's progression were evaluated in young APP/PS1 (amyloid precursor protein/presenilin 1) mice. Sleep was monitored with electroencephalography/electromyography (EEG/EMG) telemetry. Wide‐field imaging with voltage‐sensitive dyes (VSDs) was used to track sleep‐dependent brain rhythms. Multiphoton microscopy allowed assessments of amyloid plaque load and basal neuronal calcium levels. Behavioral assays were used to measure memory and cognitive function.

**RESULTS:**

Zolpidem restored NREM sleep and rescued sleep‐dependent brain rhythm, slow oscillation. Zolpidem administration reduced cortical amyloid plaque burden, mitigated neuronal calcium overload, and enhanced sleep‐dependent contextual recall without adverse effects on locomotion.

**DISCUSSION:**

Zolpidem effectively decreased amyloid in young APP/PS1 mice. This supports zolpidem's therapeutic promise as an intervention strategy at early stages of AD.

**Highlights:**

- Zolpidem treatment improves non–rapid eye movement (NREM) sleep stability and reduces sleep fragmentation.
- Zolpidem restores slow oscillation in young APP/PS1 (amyloid precursor protein/presenilin 1) mice.
- Zolpidem treatment reduces amyloid plaque burden and calcium overload in neurons.
- Zolpidem‐treated mice show improved sleep‐dependent memory consolidation.
- Sleep rhythm enhancement shows promise for Alzheimer's therapy.

## 1 BACKGROUND

Alzheimer's disease (AD) is a progressive neurodegenerative disorder and the leading cause of dementia worldwide, affecting over 55 million people globally, a number expected to double by 2050.^1^, ^2^ It is clinically characterized by progressive cognitive decline and memory impairment. AD is pathologically characterized by the accumulation of amyloid‐beta (Aβ) plaques and neurofibrillary tau tangles.^3^, ^4^

Sleep impairments have emerged as a major modifiable risk factor for Alzheimer's disease. Numerous studies have demonstrated that sleep deprivation and poor sleep quality are associated with increased amyloid‐β accumulation and accelerated disease progression.^5^, ^6^ These pathological changes are closely linked to disruptions in the sleep–wake cycle, particularly deficits in non–rapid eye movement (NREM) sleep.^7^ AD patients exhibit significant alterations in sleep architecture, including reduced NREM sleep duration, fragmented sleep, and diminished restorative sleep quality.^8^, ^9^ Among these changes, impairments in slow oscillation (0.5–1 Hz), a sleep‐dependent brain rhythm and the hallmark electrophysiological signature of deep NREM sleep, have gained increasing attention.^10^ Slow oscillation is a large‐amplitude, low‐frequency cortical rhythm that organizes synchronized neuronal activity across widespread brain networks.^11^, ^12^ Each slow oscillation cycle consists of a downstate of relative neuronal silence and an upstate of synchronous neuronal firing.^13^, ^14^, ^15^ Down and up states alternate during slow wave activity. Slow oscillation is important for restorative sleep, contributing to cognitive functions such as memory consolidation and learning in healthy individuals.^13^, ^16^ Slow oscillation supports the homeostatic regulation of sleep, increasing in response to prolonged wakefulness and decreasing throughout the sleep period. In addition, slow oscillation helps organize faster brain rhythms, including sleep spindles and sharp‐wave ripples, thereby enhancing the efficiency of neural networks involved in executive functions and synaptic plasticity.^17^, ^18^, ^19^ Our published work showed that optogenetic rescue of slow waves slowed AD, while exacerbating slow wave deficits facilitated AD in a mouse model.^20^, ^21^ Therefore, therapeutic targeting of slow oscillation could present a promising approach to slowing Alzheimer's progression.

Mechanistically, impaired inhibitory neurotransmission has been identified as a major contributor to slow wave disruptions in young APP/PS1 (amyloid precursor protein/presenilin 1; APP) mice, a mouse model of amyloidosis.^21^, ^22^ Our published studies demonstrated that enhancing gamma‐aminobutyric acid‐ergic (GABAergic) tone through exogenous GABA administration restored slow oscillation in APP mice.^21^ However, pharmacological block of GABA receptors disrupted slow oscillation in healthy mice.^21^ Similar to Alzheimer's patients, young APP mice exhibit deficits in NREM sleep and increases in wake durations. Optogenetic stimulation of GABAergic interneurons in the anterior cortex effectively rescued sleep deficits and restored slow oscillation in APP mice.^22^ These findings emphasize the importance of inhibitory circuits in maintaining healthy sleep rhythms and suggest that strategies aimed at enhancing inhibitory neurotransmission could have therapeutic benefits at early stages of AD.

Based on these insights, we performed an unbiased computational screen that identified zolpidem as high‐affinity GABA_A_ receptor modulator facilitating chloride transport that could slow AD.^23^ We adopted a translational approach by testing zolpidem, an United States Food and Drug Administration (FDA) ‐approved drug that enhances GABA_A_ receptor activity and promotes sleep.^24^ We systematically assessed the therapeutic potential of zolpidem in APP mice using multiple complementary experimental techniques. We first used in vivo voltage‐sensitive dye (VSD) imaging to directly visualize the acute effects of zolpidem on cortical slow oscillation dynamics across different doses, identifying 30 mg/kg as the optimal concentration for restoring slow oscillation. Wireless EEG/EMG telemetry was used to demonstrate that zolpidem improved NREM sleep architecture, enhancing NREM sleep continuity and stability while reducing sustained wakefulness. Importantly, chronic daily administration of zolpidem for four weeks significantly lowered amyloid plaque burden and neuronal calcium overload. Behavioral assessments revealed that zolpidem treatment selectively improved sleep‐dependent contextual recall without adversely affecting locomotor activity or working memory. Collectively, our findings suggest that zolpidem, an FDA‐approved agent, may serve as a promising therapeutic approach to restore sleep‐dependent brain rhythms and ameliorate key pathological features of Alzheimer's disease.

## 2 METHODS

### 2.1 Animals and drug administration

The mouse strain used for this research project, B6C3‐Tg (APPswe, PSEN1dE9) 85Dbo/Mmjax, RRID:MMRRC_034829‐JAX, was obtained from the Mutant Mouse Resource and Research Center (MMRRC) at The Jackson Laboratory, and National Institutes of Health (NIH) ‐funded strain repository, and was donated to the MMRRC by David Borchelt, Ph.D., McKnight Brain Institute, University of Florida. Mice were randomly assigned to the experimental and control groups, ensuring similar representation of males and females to minimize potential sex‐related confounding effects. All mice were maintained under a 12:12 hours light/dark cycle (7:00 AM lights on, 7:00 PM lights off) with ad libitum access to food and water. For acute treatment experiments, APP mice aged 3–6 months were used to assess immediate effects of zolpidem on slow oscillation and sleep architecture, both of which were impaired during these ages.^21^, ^22^, ^25^ For chronic treatment experiments, APP mice aged 7–9 months were used to evaluate the long‐term impact of zolpidem on AD‐related pathology, such as plaques and neuronal calcium overload, which are evident at these ages. Also, 7–9‐month‐old APP mice were treated with zolpidem and followed with assessments of memory function, which is impaired at these ages.^26^ Vehicle (0 mg/kg) or zolpidem (3,10, 30 mg/kg) was administered via intraperitoneal (i.p.) injection. 10% dimethyl sulfoxide (DMSO) in phosphate buffered saline (PBS) served as vehicle. The administration period and time of day for each experiment were specified in the corresponding sections. All procedures were approved by the Institutional Animal Care and Use Committee (IACUC, protocol number 2012N000085) and conducted in accordance with NIH and ARRIVE guidelines.

**RESEARCH IN CONTEXT**

1. **Systematic review**: Accumulating evidence shows that non–rapid eye movement (NREM) sleep and sleep‐dependent brain rhythm, slow oscillation (0.5–1 Hz), plays a key role in memory consolidation and amyloid‐beta (Aβ) clearance. Animal and human studies suggest NREM deficits, due to reduced inhibitory tone, contribute to Alzheimer's disease (AD) progression. Thus, computational modeling was employed to identify and repurpose United States Food and Drug Administration (FDA) ‐approved therapeutics targeting the gamma‐aminobutyric acid A (GABA_A_) receptor. Unbiased computational screen identified zolpidem as the leading compound.
2. **Interpretation**: Zolpidem, an FDA‐approved hypnotic, improves sleep architecture, enhances slow oscillation, and reduces AD‐related pathology in APP/PS1 mice. Benefits included lower amyloid burden, restored neuronal calcium balance, and improved memory, without adverse behavioral effects.
3. **Future directions**: These findings support targeting sleep‐dependent brain rhythms as a therapeutic strategy at early stages of AD. Future work should evaluate long‐term efficacy and safety of zolpidem in clinical populations and explore its synergy with other disease‐modifying therapies for AD.

### 2.2 VSD imaging

To evaluate the acute effects of zolpidem on cortical slow oscillation, wide‐field in vivo imaging was performed using the VSD RH2080. APP mice (3–6 months old) underwent craniotomy (5 mm diameter) centered over the somatosensory cortex under 1%–2% isoflurane anesthesia. RH2080 (1 mg/mL in PBS) was applied topically for 1 h. After washing off excess dye with PBS and sealing the craniotomy with a glass coverslip, imaging was performed using a Hamamatsu ORCA‐ER charge‐coupled device (CCD) camera (Hamamatsu Photonics) with a 630 ± 10 nm excitation filter and *a* > 665 nm long‐pass emission filter. Imaging was conducted during the light cycle (between 10:00 AM and 5:00 PM) under 1%–2% isoflurane anesthesia. Images were acquired at 20 Hz using Imaging Workbench software (INDEC Systems). A single dose of zolpidem (3,10, 30 mg/kg) or vehicle (0 mg/kg) was administered via intraperitoneal (i.p.) injection, followed with repeated imaging at 5‐minute intervals during the first 30 minutes postinjection, followed by longer intervals up to 120 minutes. A between‐subject design was used, with each animal assigned to either vehicle or zolpidem treatment. Data were motion‐corrected and spatially filtered before analysis. Slow oscillation power was extracted using temporal band‐pass filtering (0.5–1 Hz) and quantified across the cortical field of view.

VSD image sequences were analyzed using ImageJ. For each recording, fluorescence changes were expressed as ΔF/F_0_, where F_0_ represented the minimum fluorescence intensity within the image sequence and ΔF was the change in pixel intensity relative to F_0_. Mean ΔF/F_0_ values were extracted from a manually defined region of interest (ROI) over the somatosensory cortex. To quantify oscillatory dynamics, Fourier transform analysis was performed in MATLAB, allowing calculation of oscillation power.

### 2.3 EEG/EMG sleep recordings

APP mice (3–6 months old) were surgically implanted with wireless electroencephalography/electromyography (EEG/EMG) telemetry devices (HD‐X02, Data Sciences International, Minneapolis, MN) under isoflurane anesthesia (3% induction, 1.5% maintenance) using aseptic techniques. To adequately record sleep, anterior and posterior electrodes (two total) were implanted to monitor cortical activity across a broad region. Additionally, these sites provided sufficient skull thickness for stable electrode placement, which was important for reproducibility in small mouse brains. This configuration followed the vendor's recommended protocol and was widely used in rodent EEG studies. Telemetry transmitters were placed subcutaneously along the dorsum. The skull was exposed and cleaned, and two stainless steel screws (M06‐15‐M‐SS‐P, US Micro Screw) serving as EEG electrodes were secured through the skull to contact the dura. The anterior electrode was placed 1 mm anterior to bregma and 1 mm lateral to the midline, while the posterior electrode was positioned 3 mm posterior to bregma and 3 mm contralateral to the anterior screw. EMG leads were sutured into the bilateral nuchal muscles. Following surgery, mice were allowed to recover for 10 days before recordings began. Vehicle or zolpidem (30 mg/kg) was administered at ZT2 (2 h after lights on, ∼9:00 AM) on days 1 and 2 of recording. EEG/EMG signals were acquired using Ponemah Software v6.50 (Data Sciences International) and continuously recorded while animals were housed in individual home cages over receiver plates (RPC‐1, Data Sciences International). Signals were digitized with a bandwidth of 0.1–200 Hz and sampled at 500 Hz. EEG/EMG data, activity, and temperature was recorded for 24 h at baseline and for 48 hours following zolpidem or vehicle injection.

Sleep scoring was performed using NeuroScore Software v3.6 (Data Sciences International), which allowed fully automated sleep scoring to minimize experimenter bias as follows. Raw EEG and EMG signals were imported and bandpass filtered (EEG: 0.5–100 Hz; EMG: 10–100 Hz). Data were segmented into 10‐s epochs and classified as wake, NREM sleep, or REM sleep using the integrated Mouse Sleep Scoring module. Wakefulness was defined by high‐frequency, variable EEG activity and high EMG tone. NREM sleep was characterized by low‐frequency, high‐amplitude EEG and low EMG tone. REM sleep was identified by dominance of theta‐band activity (theta/delta power ratio > 3) and minimal EMG tone. Sleep bouts were defined as a minimum of two consecutive epochs of the same state. Power spectral density analysis was performed using fast Fourier transform (FFT) with a Hanning window applied to artifact‐free epochs. Spectral bands were defined as follows: slow oscillation (0.5–1 Hz), delta (1–4 Hz), theta (4–8 Hz), alpha (8–12 Hz), sigma (12–16 Hz), and beta (16–24 Hz). Relative band power was computed as the ratio of individual band power to total power. Changes in sleep architecture and spectral composition were assessed across baseline and post‐treatment periods.

### 2.4 Multiphoton imaging of amyloid and calcium

To monitor AD‐related pathology in vivo, we performed longitudinal two‐photon imaging of amyloid plaques and neuritic calcium using Methoxy‐X04 and the genetically encoded ratiometric calcium indicator Yellow Cameleon 3.6 (YC3.6).^27^ Vehicle or zolpidem (30 mg/kg) were administrated at ZT2 (2 hours after lights on, ∼9:00 AM) for 4 weeks of daily treatment.

### 2.5 AAV‐YC3.6 viral injection

Mice were initially anesthetized with 5% isoflurane in oxygen and maintained at 1.5% isoflurane throughout the surgical procedure. Body temperature was maintained at approximately 37.5°C using a heating pad, and ophthalmic ointment was applied to protect the eyes. Using sterile techniques, a 5 mm diameter round craniotomy was performed over the somatosensory cortex, alternating between the right and left hemispheres across animals to minimize lateralization bias.

To visualize basal neuronal calcium levels, AAV2‐CBA‐YC3.6 (2 µL, 2 × 10^1^ ^2^ vg/mL; University of Pennsylvania Vector Core) was injected bilaterally into the posterior somatosensory cortices of 7‐month‐old APP mice. Stereotaxic coordinates relative to bregma were: anterior–posterior (AP) −1.5 mm, medial–lateral (ML) ± 1.5 mm, dorsal–ventral (DV) −0.8 mm. The virus was delivered at a rate of 0.15 µL/min via a pulled glass micropipette, which was left in place for an additional 10 minutes postinjection to facilitate viral diffusion. Following viral injection, a sterile glass coverslip was positioned over the craniotomy and secured using dental cement to form a chronic cranial window. The skin was sutured around the window margin. Mice were allowed to recover on a heating pad until fully awake and mobile and were subsequently monitored daily. Experimental imaging began three weeks post‐surgery to ensure complete recovery and sufficient transgene expression.

#### 2.5.1 Multiphoton imaging of amyloid plaques

To visualize amyloid plaque pathology in vivo, two‐photon imaging was performed through chronic cranial windows in mice. One day prior to imaging, mice received an intraperitoneal injection of Methoxy‐X04 (10 mg/kg, Tocris), which binds to fibrillar amyloid and enables fluorescence‐based detection. Immediately before imaging, Texas Red dextran (70 kDa, 2.5% in PBS; Thermo Fisher) was administered intravenously via the retro‐orbital sinus to label the cerebral vasculature and facilitate anatomical orientation. Imaging was conducted using a FluoView FV1000MPE two‐photon laser‐scanning imaging system (Olympus) mounted on a BX61WI upright microscope with a 25× long working distance water‐immersion objective (NA = 1.05, Olympus). A mode‐locked Ti:Sapphire laser (MaiTai, Spectra‐Physics) was tuned to 800 nm to excite Methoxy‐X04. Images were acquired at 1× digital magnification, and Z‐stacks were collected at 5 µm intervals. Body temperature was maintained using a heated stage, and mice remained under 0.5–1% isoflurane anesthesia during acquisition.

#### 2.5.2 Multiphoton imaging of calcium

To acquire basal neuronal calcium levels in APP mice, imaging of ratiometric YC3.6‐expressing neuronal processes (neurites) was performed through the same cranial windows as imaging of amyloid plaques. APP mice were injected with AAV2‐CBA‐YC3.6 into the somatosensory cortex three weeks prior to imaging as described above (2.4.1). Imaging was performed on the same two‐photon system using a Ti: Sapphire laser tuned to 860 nm to excite YC3.6. Fluorescence signals were separated using a 510 nm dichroic mirror and collected via PMTs set to detect cyan fluorescent protein (CFP) (380–480 nm) and yellow fluorescent protein (YFP) (500–540 nm). ROIs were selected based on morphology and expression clarity. Digital magnification was set to 2×. To minimize photodamage, laser power was maintained below 30 mW. Z‐stack images were acquired at 1 µm intervals. Ratiometric (YFP/CFP) images were generated pixel‐wise, and calcium‐overloaded neurites were identified as those with mean YFP/CFP ratios exceeding 1.79, indicating cytosolic calcium levels > 235 nM.

### 2.6 Image analysis

Z‐stacks were aligned to baseline using rigid‐body registration (TurboReg, ImageJ). Methoxy‐X04–labeled amyloid plaques were segmented using intensity‐based thresholding and reconstructed in 3D using Imaris (Bitplane). Total plaque burden was quantified as the sum of all segmented plaque volumes per field of view, and change over time was expressed as percent of baseline.

YC3.6‐expressing neurites were manually segmented, and pixel‐wise ratiometric YFP/CFP images were generated. For each animal, multiple neurites were sampled per imaging session. A neurite was defined as having calcium overload if its mean YFP/CFP ratio exceeded 1.79, corresponding to intracellular calcium levels > 235 nM. The proportion of overloaded neurites was calculated per mouse and used as the primary outcome measure of treatment efficacy.

### 2.7 Behavioral testing

All behavioral experiments were conducted in a new cohort of mice that did not undergo cranial window or EEG/EMG probe surgeries. This approach was chosen to eliminate potential confounding effects of surgical manipulations on behavioral outcomes. Vehicle or zolpidem (30 mg/kg) were administrated at ZT2 (2 h after lights on, ∼9:00 AM) for 4 weeks of daily treatment.

#### 2.7.1 Open field test

Mice were individually placed in a 27 × 27 × 20 cm acrylic arena and allowed to explore freely for 10 min. Locomotion was tracked via video recording and analyzed with EthoVision XT software (Noldus). Total distance traveled, time spent in the center zone, and center entry frequency were quantified to evaluate locomotor activity and anxiety‐like behavior.

#### 2.7.2 Y‐Maze test

Each mouse was introduced to the center of a Y‐maze (arm dimensions: 5 × 30 × 12 cm; arms arranged at 120° angles) for a 10‐min exploration period. An arm entry required all four limbs to cross into an arm. Spontaneous alternation (SA) percentage was calculated as SA% = [Number of triads / (Total entries − 2)] × 100, with a triad defined as consecutive entries into three different arms. The maze was disinfected with 70% ethanol between tests.

#### 2.7.3 Fear conditioning test

Training took place in chambers (30 × 24 × 21 cm; MED‐Associates) equipped with a stainless‐steel grid floor and auditory stimulation system. Following a 2‐min habituation, mice received three tone‐shock pairings (80 dB, 30‐seconds tone; 0.6 mA shock during the final 2 seconds, co‐terminating with the tone), separated by 210‐s intervals. Training was conducted between 5:00 and 7:00 PM.

For contextual recall, mice were returned to the same chambers 24 h later and observed for 3 min without tone or shock. One hour later, cued recall was assessed in a modified environment with altered floor and wall features. After 3 min of exploration, a 30‐s tone was presented without shock. Freezing behavior was automatically analyzed using Video Freeze software (Med Associates Inc.). Chambers were sanitized with 70% ethanol between sessions.

### 2.8 Computational methods

#### 2.8.1 High‐throughput virtual screening

For protein–ligand docking analysis, 3D structure of intact GABA_A_ receptor unit was retrieved from PDB databank (https://www.rcsb.org/) with accession ID 6D6U.^28^ FDA‐approved drug library was retrieved from DrugBank database (https://go.drugbank.com/). GABA_A_ receptor structure was prepared before docking analysis, using Protein Preparation Wizard from Schrodinger Maestro SBDD suite. Since the retrieved structure has a bound ligand (flumazenil), before screening, a short simulation for 100 ns was conducted without any intact ligand, using Desmond simulation package to relax the protein complex. Retrieved FDA‐approved drug library was prepared using LigPrep module from Schrodinger Maestro SBDD suite. For high‐throughput virtual screening, Glide algorithm from Schrodinger Maestro SBDD suite was used.^29^ Docking grid‐box was generated based on the information from bound ligand (flumazenil) in the retrieved PDB structure (6D6U). The first stage docking was conducted using standard precision mode, followed by high‐precision mode. For Gibbs binding free energy, MMGBSA method was used via Schrodinger Prime module.^29^

#### 2.8.2 Machine learning and neural network analysis

For predicting GABA_A_ receptor agonists and antagonists via ML approach, known GABA_A_ receptor agonists and antagonists were retrieved from PubChem database as smiles format and converted to SDF format using OpenBabel. Molecular descriptors (both 2D and 3D) for the retrieved compounds were calculated using QikProp module from Schrodinger SBDD suite.^30^ Machine learning (ML) and neural network analysis were performed using Orange data mining software.^31^, ^32^

#### 2.8.3 Molecular dynamic simulation

Molecular dynamic (MD) simulations were performed using Desmond simulation package.^33^, ^34^ Protein‐ligand complex was first prepared using Protein Preparation Wizard from Maestro Visualizer. To mimic the physiological conditions, MD simulations of GABA_A_ receptor::drug complex were performed in a virtual lipid bilayer (cell membrane) system. GABA_A_ receptor::drug complex was placed in a POPC lipid bilayer system in such a way that the lipid bilayer covers the transmembrane region of GABA_A_ receptor. This GABA_A_ receptor::drug complex with intact lipid bilayer was then immersed in a triclinic simulation box containing Simple Point Charge (SPC) water. Simulation system was filled with 0.15 M NaCl, excluding the counterions added to neutralize the system. After energy minimization (5000 steps) and equilibration (300 ps), the whole system was simulated for 200 ns. Simulation trajectories were analyzed for movement of chloride ions in and out of the membrane pore present at the M2 transmembrane region of GABA_A._

### 2.9 Statistical analyses

Statistical analyses were performed using GraphPad Prism 9 and R (R Foundation for Statistical Computing). Data were first tested for normality (Shapiro–Wilk test). Depending on distribution, comparisons were made using parametric (t‐test and analysis of variance [ANOVA] with Tukey's) or non‐parametric (Mann–Whitney U test, Kruskal–Wallis test followed by Dunn's multiple comparisons test). To control the family‐wise Type I error rate across these three planned comparisons, Bonferroni correction was applied. Results were presented as mean ± SEM unless otherwise specified. *P*‐values < 0.05 were considered statistically significant. All analyses were conducted with the experimenter blinded to treatment group.

## 3 RESULTS

### 3.1 Computational modeling identified structural dynamics of ligand binding to GABA_A_ receptor

Young APP mice exhibit NREM sleep deficits and impairments of slow oscillation due to deficits in GABA and GABA receptor expression.^21^ Thus, we set out to perform an unbiased computational screen to identify an FDA‐approved compound that could potentiate GABA_A_ signaling, rescue sleep and slow AD.

**FIGURE 1:**
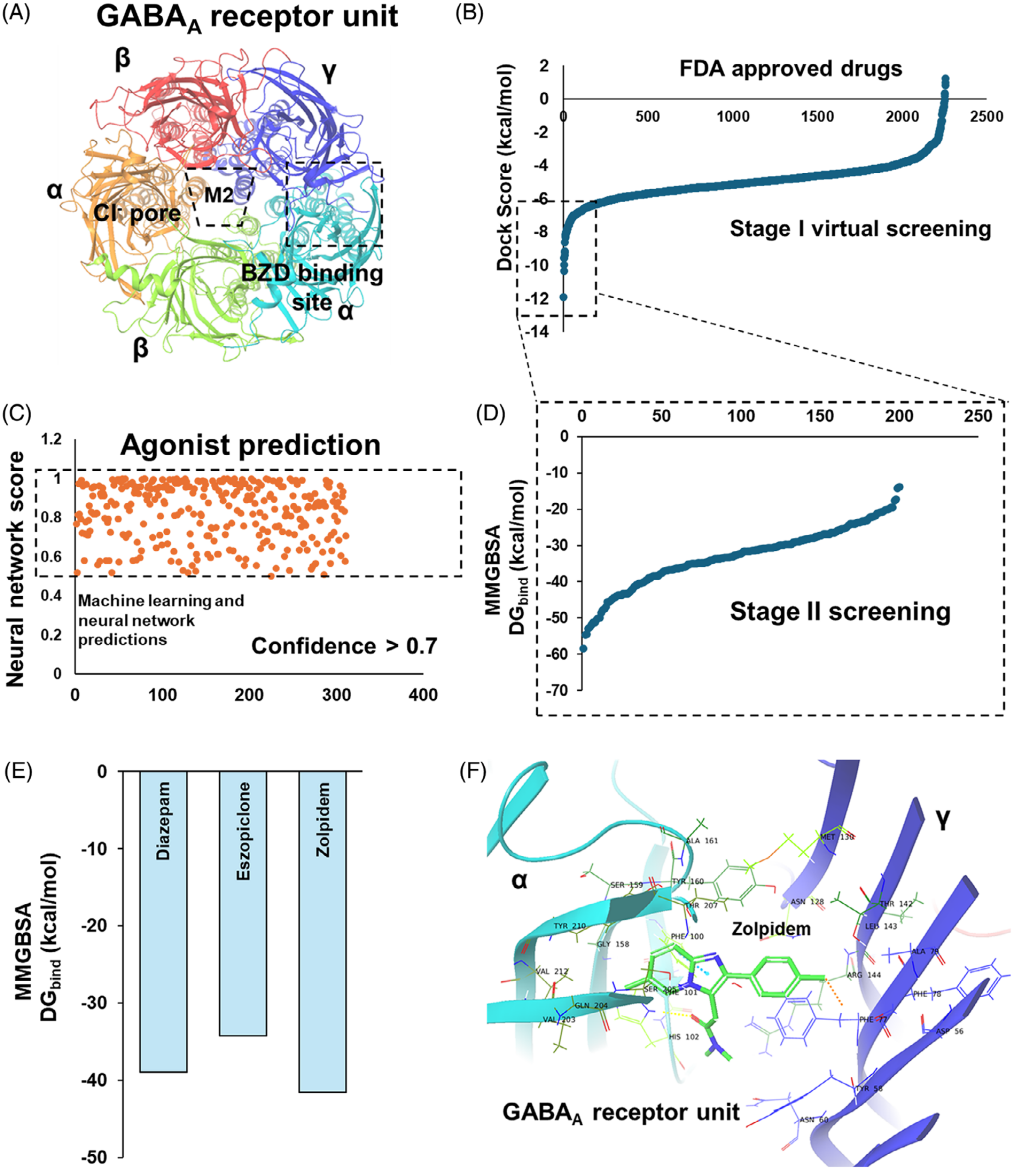
Quaternary structure and functional dynamics of the GABA_A_ receptor. (A) Experimental structure of the GABA_A_ receptor showing distinct subunits (colored based on GABA_A_ receptor subunits, BZD, α, β, and γ), forming the quaternary assembly. The ligand‐binding site, BZD binding site, is delineated with a box. (B) Predicted docking scores for the FDA‐approved drug library, ranked from highest to lowest interaction energies during Stage I of the screen. (C) Machine learning and neural network‐based classification of the top 300 compounds from (B), distinguishing predicted GABA_A_ receptor agonists from antagonists. (D) Predicted MM‐GBSA–based Gibbs binding free energies for the top 200 compounds identified in (B) during Stage I of the screen. (E) Comparison of predicted Gibbs binding free energies for zolpidem and eszopiclone with that of diazepam. (F) Predicted binding pose of zolpidem in complex with the GABA_A_ receptor, highlighting interacting residues within the BZD binding site. BZD, benzodiazepine; FDA, United States Food and Drug Administration; GABA_A_, gamma‐aminobutyric acid A; MMGBSA, Molecular Mechanics‐Generalized Born Solvent Accessible.

To investigate the structural dynamics involved in ligand binding to the GABA_A_ receptor, the three‐dimensional quaternary structure of the receptor was retrieved from the Protein Data Bank (PDB). The structure comprises two α subunits, two β subunits, and one γ subunit. Structural visualization revealed the presence of a membrane pore (M2) and the benzodiazepine (BZD) binding site (Figure 1A). The BZD binding site is located at the interface between the α and γ subunits of the GABA_A_ receptor (Figure 1A). Since both GABA_A_ receptor agonists and antagonists primarily target the BZD binding region,^28^, ^35^ docking calculations were performed using known positive allosteric modulators (PAMs), for example, diazepam, and competitive antagonists, for example, flumazenil (FYP) as standard ligands targeting the BZD binding site. The term “agonist” here refers to PAMs that enhance GABA inhibitory action. The term “antagonist” refers to competitive antagonists, drugs that inhibit GABA inhibitory action. Docking results predicted binding affinities of –38.91 kcal/mol for diazepam and –27.56 kcal/mol for flumazenil at the BZD site (Supplementary Figure 1A).

To further understand the dynamics of ligand binding, 200 ns atomistic MD simulations were performed on the docked complexes of the GABA_A_ receptor bound to either diazepam or flumazenil, with the receptor embedded in a virtual lipid membrane (Supplementary Figure 1B). Since the chloride transport through the M2 membrane pore is one of the key functional properties of the GABA_A_ receptor, the number of chloride ions passing through the M2 pore during the simulations was analyzed. Diazepam facilitated chloride ion transport across the membrane throughout the 200 ns simulation (Supplementary Figure 1C). In contrast, flumazenil inhibited chloride ion transport across the membrane when bound to the GABA_A_ receptor (Supplementary Figure 1D).

### 3.2 High‐throughput virtual screening and neural network predicted high‐affinity GABA_A_ receptor modulators

To identify drug molecules targeting the GABA_A_ receptor as potentiators, we computationally screened an FDA‐approved drug library against the BZD‐binding site, based on the docking models as shown in Figure 1B. The docking protocol was conducted in two stages. In Stage I, a high‐throughput (standard‐precision) docking approach was used to screen approximately 2,300 FDA‐approved drugs against the GABA_A_ receptor. Drug candidates were ranked based on docking scores (Figure 1B). Since both agonists and antagonists bind at the BZD‐binding site, ML and neural network models were employed to classify potential agonists among the top‐ranked compounds. For model training, we curated a dataset of 32 known GABA_A_ receptor agonists and 24 antagonists from public sources, including the PubChem database. Multiple ML algorithms were evaluated, including Support Vector Machine (SVM), Logistic Regression, Random Forest, and Neural Networks. Molecular descriptors (∼277) were computed for all compounds, both training and unknown, using the Mordred tool. Model development, training, and predictions were conducted using Orange Data Mining.

Model performance was assessed via random sampling. Among the tested models, SVM and Neural Networks showed the highest prediction accuracies, with SVM achieving 75.3% accuracy (area under the curve [AUC]: 80.3%) and the Neural Network model achieving 74.4% accuracy (AUC: 77.3%). Other models, including Logistic Regression, showed accuracies below 70% and were therefore excluded from further analysis. Model training was repeated multiple times to ensure robustness, with SVM and Neural Network consistently demonstrating strong predictive performance. Once trained, these models were used to predict the agonist/antagonist classification for the full set of 2,256 FDA‐approved therapeutics during Stage I. To minimize false positives, only compounds predicted as agonists by both the SVM and Neural Network models were selected, yielding 311 candidates (Figure 1C).

To further refine the predictions, the top 200 compounds (top 10% from Stage I) were subjected to Stage II screening using the Molecular Mechanics Generalized Born Solvent (MM‐GBSA) method. Drug candidates were ranked based on estimated Gibbs free energy of binding (Figure 1D). Using predictions from ML (properties) and computational docking (binding affinity) estimates (Figure 1E), combined with its hypnotic properties, Zolpidem was selected as the leading drug candidate for further investigation.

### 3.3 MD simulation predicted stability in zolpidem's binding to GABA_A_ receptor and facilitating chloride ion transport

Protein–ligand docking analysis of the GABA_A_ receptor bound to target drug molecules identified key amino acid residues involved in ligand recognition. Diazepam interacted with γPhe77, α1Thr162, α1Ser204, and α1Ser206 (Supplementary Figure 2A,B). Protein–ligand interaction profiling of GABA_A_ receptor bound to zolpidem showed similar interaction pattern at the BZD‐binding site when compared to diazepam (Figure 1F). Structural superimposition of predicted binding pose for zolpidem and diazepam showed zolpidem bound to the same facet as diazepam (Supplementary Figure 2G).

To evaluate the binding stability of zolpidem and its potential to facilitate chloride ion transport across the membrane, a 200 ns MD simulation of the GABA_A_ receptor–zolpidem complex was performed, with the receptor embedded in a virtual lipid bilayer. Simulation trajectories were analyzed to assess binding stability and the number of contacts between chloride ions and the M2 transmembrane region (chloride pore) of the receptor. Zolpidem maintained stable binding at the BZD site throughout the 200 ns simulation (Supplementary Figure 3A).

Snapshots from simulation trajectories at different time points revealed that zolpidem binding induced structural changes in the GABA_A_ receptor, notably the formation of a membrane pore (Supplementary Figure 3B). In contrast, competitive antagonist flumazenil inhibited such membrane pore formation during the 200 ns simulation (Supplementary Figure 3C).

Since zolpidem induced a membrane pore formation, we next examined whether it could facilitate chloride ion transport. Analysis of the simulation trajectories for the number of chloride ions passing through M2 membrane showed that zolpidem facilitated chloride ion transport across the membrane, with an increased number of chloride ions passing through the M2 membrane pore over the course of the 200 ns simulation (Supplementary Figure 4A, B). Visualization of chloride ion trajectories indicated Cl^−^ ions traversed the virtual membrane from the extracellular side to the cytoplasmic side, interacting with M2 transmembrane residues (Supplementary Figure 4A). Thus, as a result of unbiased computational screen, zolpidem emerged as a leading candidate potentiating GABA_A_ signaling. Zolpidem's effect on slow oscillation, sleep and amyloidosis was tested in a mouse model of amyloidosis, young APP mice.

### 3.4 Zolpidem restored slow oscillation in APP mice

**FIGURE 2:**
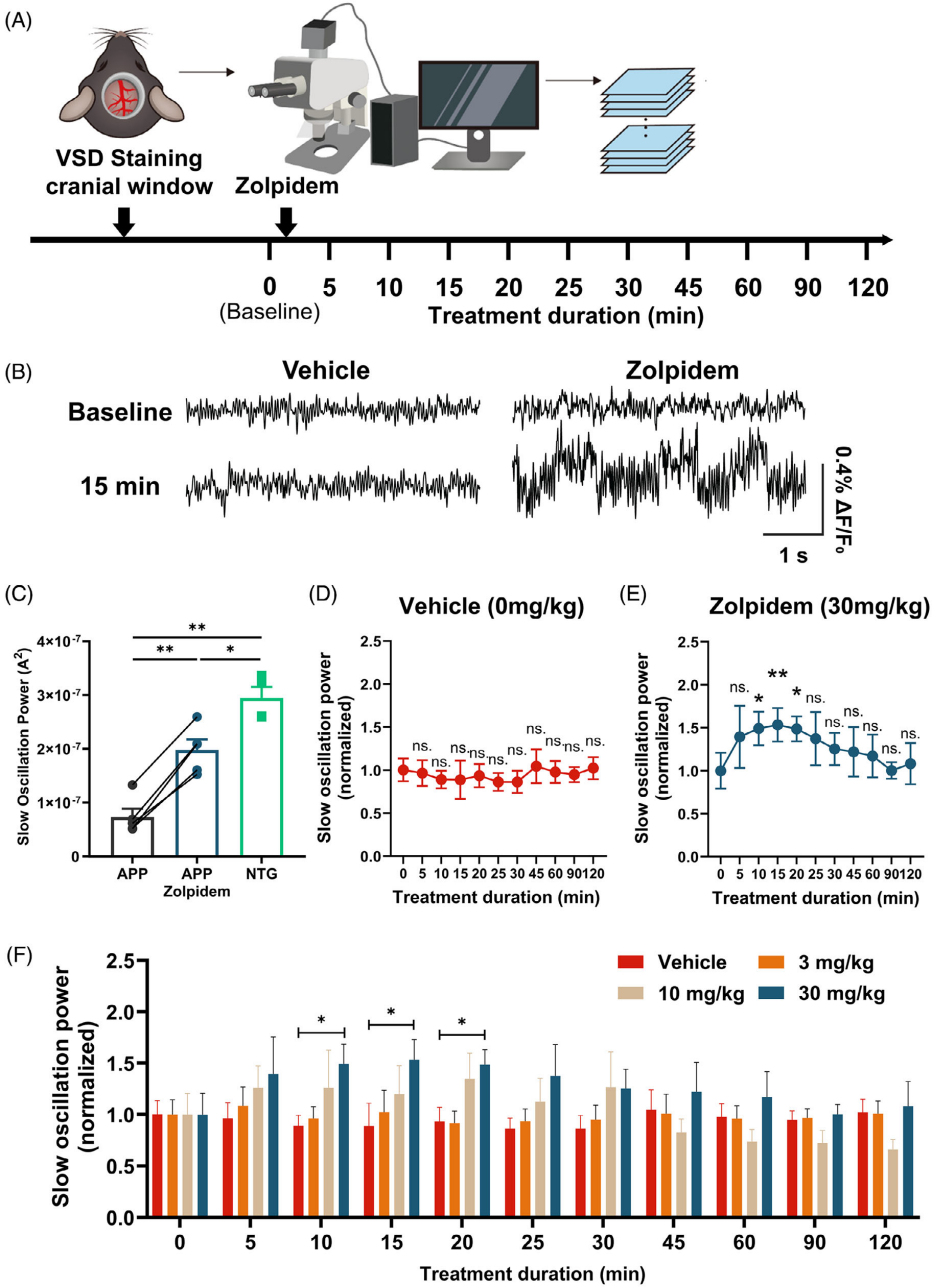
VSD imaging revealed zolpidem‐induced enhancement of cortical slow oscillation. (A) Schematic of VSD imaging setup over somatosensory cortex in an APP mouse. (B) Sample VSD imaging traces from cortical regions of interest showing slow oscillation in anesthetized APP mice treated with vehicle (0 mg/kg) or zolpidem (30 mg/kg) at baseline and 15 min after treatment. (C) Bar graph comparing slow oscillation powers across groups in APP mice at baseline (APP), APP mice 20 minute after zolpidem treatment (APP zolpidem) and untreated NTG. Each data point represents the average of 10–15 traces from each mouse. APP and APP zolpidem data were obtained from the same mice before and after treatment; therefore, the data were analyzed using a paired two‐tailed t‐test to evaluate within‐subject differences (APP vs. APP zolpidem; **p = 0.0031). NTG group consisted of a separate set of animals; thus, NTG data were compared to each treatment condition (NTG vs. APP; **p = 0.0015, and NTG vs. APP zolpidem; *p = 0.035) using Welch's unequal‐variance t‐tests. Bonferroni correction was applied to control the family‐wise Type I error rate across the three comparisons. (D) Statistical comparison of slow oscillation (0.5–1 Hz) power before and after vehicle (0 mg/kg) treatment. Friedman test followed by Dunn's multiple comparisons test. (E) Statistical comparison of slow oscillation (0.5–1 Hz) power before and after 30 mg/kg zolpidem treatment. Friedman test followed by Dunn's multiple comparisons test. (F) Time course of normalized slow oscillation power across 120 min postinjection in vehicle (0 mg/kg), 3 mg/kg, 10 mg/kg, and 30 mg/kg treated groups. Slow oscillation power was normalized to each animal's baseline (pre‐treatment, *x* = 0 minute). Statistical analyses were conducted using a one‐way ANOVA followed by Tukey's post hoc multiple comparisons tests. Data are shown as mean ± SEM (*n* = 4–5 mice/group). *p < 0.05, **p < 0.01. n.s., non‐significant. Lack of stars indicates lack of significance. ANOVA, analysis of variance; APP, amyloid precursor protein; NTG, nontransgenic controls; VSD, voltage‐sensitive dye.

APP mice exhibit slow oscillation of low power compared to that of nontransgenic littermate controls.^22^ To determine the extent to which zolpidem restores slow oscillation in APP mice, we first recorded baseline cortical slow oscillation using VSD through cranial windows via wide‐field microscopy in vivo (Figure 2A). We confirmed that slow oscillation power was low in APP mice compared to that of nontransgenic mice under the current experimental settings (Figure 2C). Zolpidem at three different concentrations (3, 10, 30 mg/kg) or vehicle control (0 mg/kg) was administered intraperitoneally (i.p.) to APP mice (*n* = 4–5 mice per group). Given the rapid permeability of zolpidem across the blood‐brain barrier,^36^, ^37^ recordings were taken at 5‐min intervals within the first 30 min postinjection, and at longer intervals up to 120 min postinjection. The quantification of slow oscillation power was derived from FFT analysis of the VSD signals. We observed a gradual increase in slow oscillation power after zolpidem administration, reaching a significant elevation relative to baseline at 10, 15, and 20 min with a 30 mg/kg dose (Figure 2B, E). Zolpidem rescued slow oscillation power to ∼50% of that of nontransgenic mice (Figure 2C). Subsequently, slow oscillation power gradually declined, returning to baseline levels (Figure 2E). Meanwhile, APP mice treated with vehicle control showed no significant changes in slow oscillation power throughout the 120‐min recording period following injection (Figure 2D). When directly compared with vehicle‐treated controls, APP mice treated with 30 mg/kg zolpidem exhibited significantly enhanced slow oscillation power at 10, 15, and 20 min postinjection (Figure 2F). However, lower concentrations of zolpidem (3 and 10 mg/kg) did not produce significant changes in slow oscillation power compared to baseline or the vehicle group (Supplementary Figure 5). Therefore, we selected 30 mg/kg zolpidem for subsequent experiments.

### 3.5 Zolpidem ameliorated NREM sleep deficits in APP mice

**FIGURE 3:**
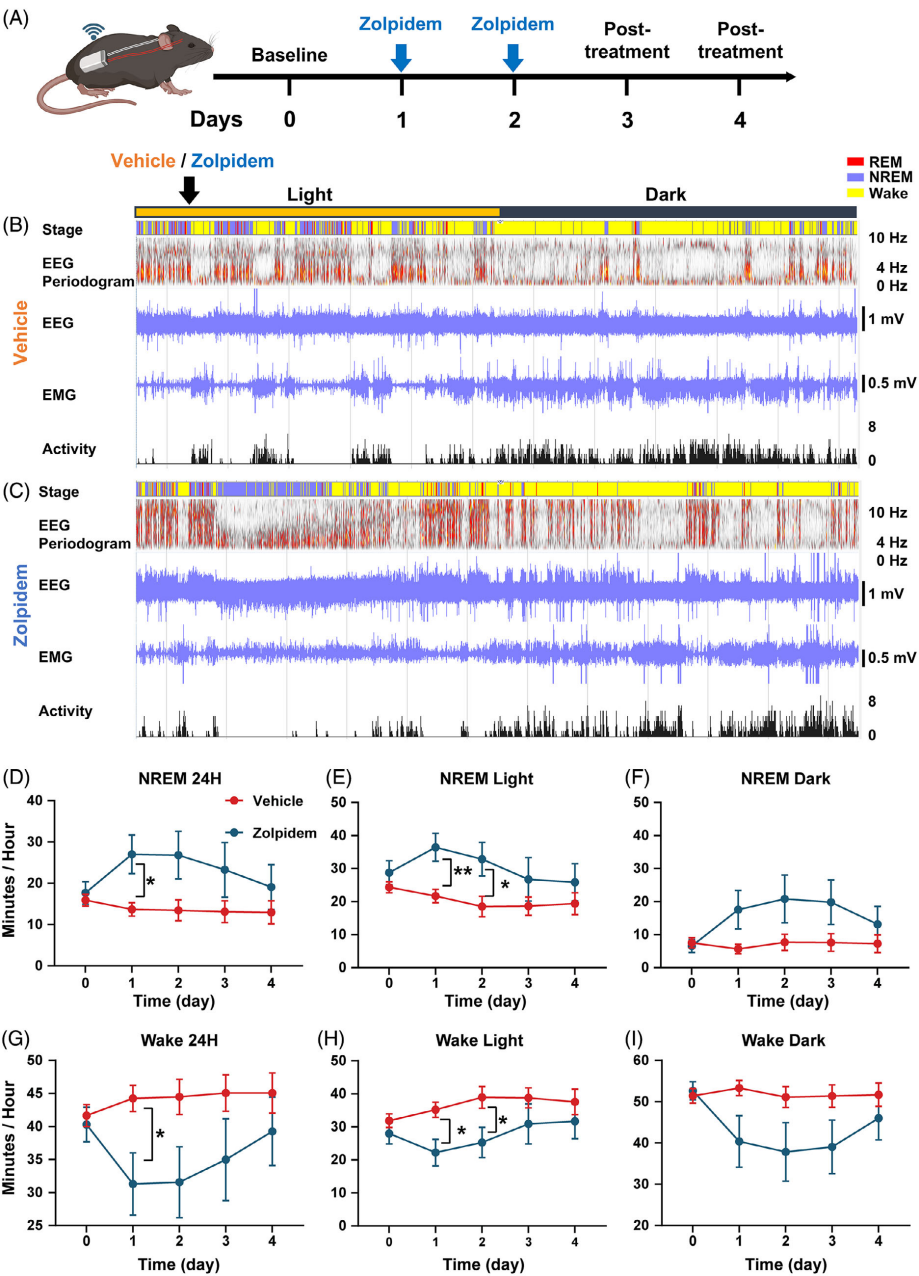
Zolpidem acutely increased NREM sleep and reduced wakefulness in APP mice. (A) Experimental timeline for EEG/EMG recordings showing baseline, zolpidem administration on days 1 and 2, and post‐treatment recording on days 3 and 4. (B) Representative hypnogram, EEG spectrogram, EEG/EMG traces, and locomotor activity for a vehicle‐treated mouse. (C) Corresponding plots for a zolpidem‐treated mouse, demonstrating increased NREM sleep (blue) and reduced wakefulness (yellow) during the light phase. (D–F) Quantification of NREM sleep durations across 24 hours (D), light phase (E), and dark phase (F). (G–I) Quantification of wakefulness durations across 24 hours (G), light phase (H), and dark phase (I). Mixed‐effects analysis followed by Tukey's multiple comparisons test. Black* indicates significant group differences between zolpidem and vehicle treatments. Data are shown as mean ± SEM (*n* = 6–7 mice/group). *p < 0.05; **p < 0.01. Lack of stars indicates lack of significance. EEG/EMG, electroencephalography/electromyography; NREM, non–rapid eye movement.

APP mice spend less time in NREM sleep and more time awake compared to nontransgenic littermate controls at 6 months of age.^22^ To determine whether zolpidem could rescue NREM sleep deficits in APP mice, we surgically implanted wireless EEG/EMG probes and performed recordings to monitor sleep architecture in freely moving animals (Figure 3A). 3‐ to 6‐month‐old APP mice were randomly assigned into one of two groups: vehicle‐treated controls or mice receiving 30 mg/kg zolpidem consecutively for 2 days. Baseline sleep patterns were recorded on day 0 prior to intervention. On day 1 and day 2, animals received one injection per day at around ZT2 (2 h after lights‐on, corresponding to 9 AM) (Figure 3B, C). EEG/EMG recordings were also taken for 2 days postinjection, on day 3 and day 4, to monitor the lasting effects of the drug.

Compared to vehicle‐treated APP mice, zolpidem treatment significantly increased NREM sleep duration and reduced wakefulness during the 24 h (Figure 3D, G, Supplementary Figure 6). That effect was prevalent during the light phase, when mice spent more time in NREM sleep (Figure 3E, H, Supplementary Figure 6). Zolpidem significantly increased NREM sleep during the light phase (Figure 3E, H). A similar trend was observed in the dark phase (Figure 3F, I), however, it did not reach statistical significance. The sleep‐inducing effects of zolpidem disappeared as early as day 3, the day after the final injection (Figure 3E, H). These findings suggest that the effects of zolpidem were short‐lived, and zolpidem had limited residual impact. On the other hand, although REM sleep duration was not consistently altered across all time points, zolpidem treatment increased REM sleep duration on day 1. No significant group differences in REM sleep were observed at later time points (Supplementary Figure 7). We also analyzed individual data points for female and male mice (Supplementary Figure 8). The distributions overlapped substantially, and no sex‐dependent differences were detected during the sleep recordings. These findings indicate that zolpidem restored NREM sleep deficits in APP mice and did not significantly interfere with the active periods.

### 3.6 Zolpidem enhanced slow oscillation and dynamically modulated high‐frequency rhythms in APP mice

**FIGURE 4 alz71175-fig-0004:**
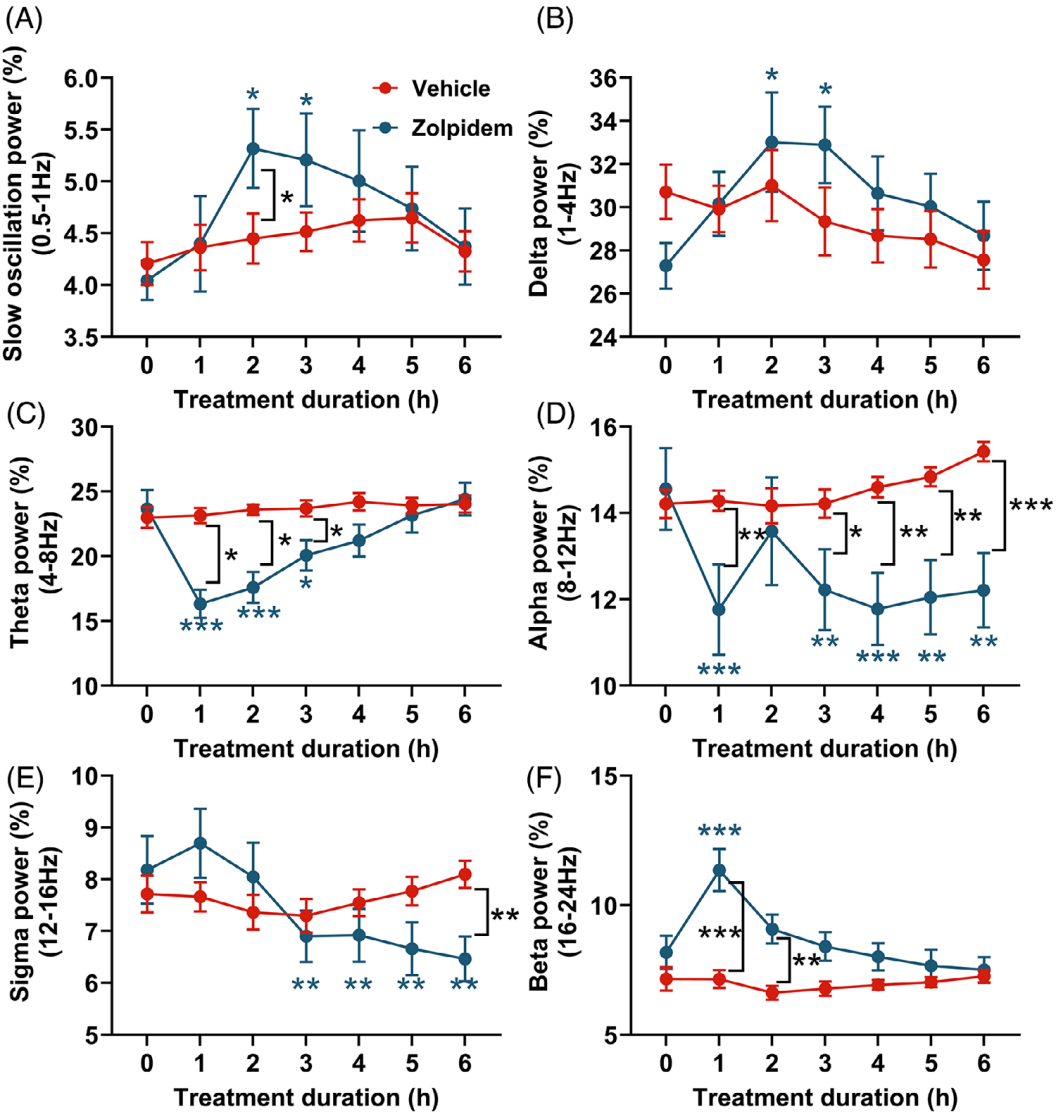
Zolpidem dynamically modulated cortical oscillatory activity across multiple frequency bands in APP mice during NREM sleep. We analyzed EEG power during NREM sleep within the first 6 hours after the drug was administered and presented the hourly changes. (A) Slow oscillation power (0.5–1 Hz) across treatment groups. (B) Delta power (1–4 Hz) across treatment groups. (C) Theta power (4–8 Hz) across treatment groups. (D) Alpha power (8–12 Hz) across treatment groups. (E) Sigma power (12–16 Hz) across treatment groups. (F) Beta power (16–24 Hz) across treatment groups. Two‐way ANOVA followed by Tukey's multiple comparisons test. Blue* indicates significant differences compared to baseline within the zolpidem group. Black* indicates significant group differences between zolpidem and vehicle treatments. Data are shown as mean ± SEM (*n* = 6–7 mice/group). *p < 0.05; **p < 0.01; ***p < 0.001. Lack of stars indicates lack of significance. ANOVA, analysis of variance; APP, amyloid precursor protein; NREM, non–rapid eye movement; EEG, electroencephalography.

Our published findings demonstrate that young APP mice exhibit reduced slow oscillation power (0.5–1 Hz) and elevated higher‐frequency components during sleep, indicative of cortical hyperexcitability, an aberration commonly observed in young amyloidosis models.^22^ To further investigate the spectral dynamics following zolpidem administration, we analyzed EEG power within the first 6 h after the drug was administered. This window captured the immediate pharmacological effects of zolpidem during the period when mice are naturally more likely to sleep. As shown in Figure 4A and B, compared to their own baseline, zolpidem treatment significantly increased slow oscillation (0.5–1 Hz) and delta (1–4 Hz) power during the first 2–3 hours postinjection. This reflected enhanced low‐frequency activity associated with deep NREM sleep. Vehicle‐treated mice showed no significant change from baseline at any time point. Moreover, direct comparisons between groups revealed that the slow oscillation power was significantly higher in the zolpidem group than in the vehicle group at 2 h postinjection (Figure 4A).

For higher‐frequency bands, zolpidem significantly suppressed theta power (4–8 Hz) during 1–3 hours postinjection compared to baseline (Figure 4C). Alpha power (8–12 Hz) was suppressed 1, 3–6 hours postinjection compared to baseline (Figure 4D). Sigma (12–16 Hz) activity was suppressed 3–6 hours postinjection relative to baseline (Figure 4E). The vehicle group showed no significant changes from baseline in these bands. Importantly, zolpidem‐treated mice exhibited significantly lower power than vehicle‐treated mice at hours 1–3 for theta, hours 1, 3–6 for alpha, hour 6 for sigma (*p* < 0.05) (Figure 4C‐D). Thus, zolpidem facilitated lower brain rhythms and effectively suppressed higher rhythms. In contrast, beta power (16–24 Hz) power transiently increased in the zolpidem group during the first hour, relative to baseline and vehicle controls (*p* < 0.05), but returned to baseline thereafter (Figure 4F). Together, these results indicate that zolpidem alters the spectral profile in APP mice by promoting slow oscillatory activity while attenuating higher‐frequency rhythms in a time‐dependent manner.

### 3.7 Chronic zolpidem treatment suppressed amyloid deposition in APP mice

**FIGURE 5 alz71175-fig-0005:**
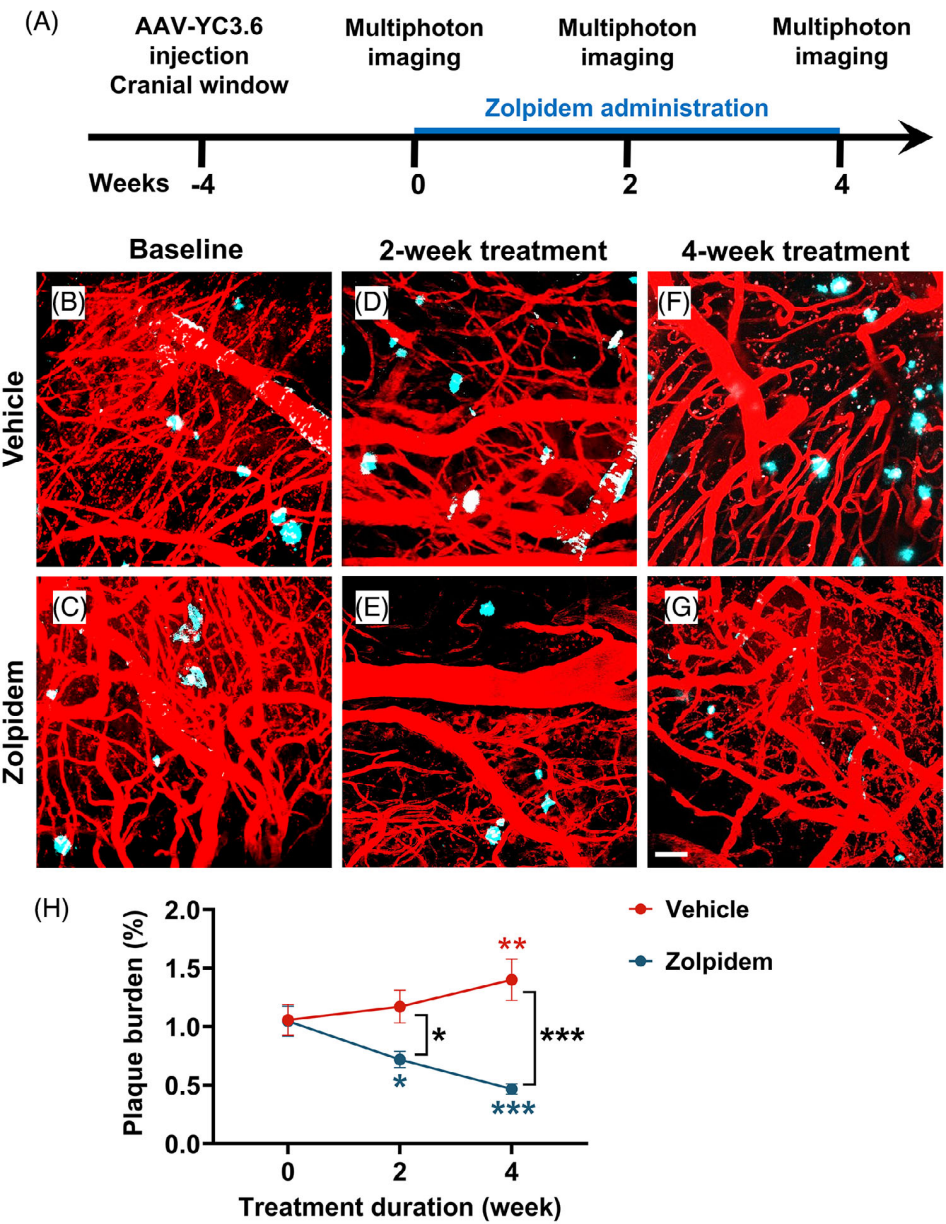
Chronic zolpidem treatment reduced amyloid plaque burden in APP mice. (A) Experimental timeline of multiphoton imaging at baseline, 2 weeks, and 4 weeks of treatment. (B–G) Representative two‐photon images of amyloid plaques (cyan, Methoxy‐X04 labeling) and cortical vasculature (red, Texas Red dextran) at baseline (B, C), after 2 weeks of treatment (D, E), and after 4 weeks of treatment (F, G) for vehicle (B, D, F) and zolpidem‐treated mice (C, E, G). Zolpidem treatment visibly reduced plaque accumulation over time. (H) Quantification of cortical plaque burden over the 4‐week treatment period. Zolpidem significantly reduced amyloid plaque burden compared to baseline and vehicle‐treated controls. Mixed‐effects analysis followed by Tukey's multiple comparisons test. Blue* indicates significant differences compared to baseline within the zolpidem group. Red* indicates significant differences compared to baseline within the vehicle group. Black* indicates significant group differences between zolpidem and vehicle treatments. Data are shown as mean ± SEM (*n* = 6–7 mice/group). *p < 0.05; **p < 0.01; ***p < 0.001. APP, amyloid precursor protein.

Building on the sleep‐enhancing effects of acute zolpidem treatment, we next sought to examine whether sustained treatment could affect AD pathology. Given the causal link between NREM sleep impairments and accelerated Aβ deposition,^38^ we assessed the impact of chronic daily zolpidem administration at 30 mg/kg on amyloid burden at baseline, weeks 2 and 4 of a continuous 4‐week treatment period (Figure 5A).

To visualize amyloid plaques in vivo over time, amyloid deposits were labeled with Methoxy‐X04. Plaques were visualized in anesthetized mice through cranial windows using multiphoton microscopy. Baseline imaging (Figure 5B, C) was performed before the start of treatment, followed by repeated imaging of same animals at weeks 2 (Figure 5D, E) and 4 (Figure 5F, G). Plaque burden was comparable between groups at baseline. In vehicle‐treated APP mice, plaque burden gradually increased, with a significant elevation at week 4 compared to baseline. In contrast, zolpidem‐treated mice exhibited a substantial reduction in plaque burden beginning at week 2, which continued to decline through week 4. A significant difference in plaque burden between zolpidem‐ and vehicle‐treated mice was observed at week 2 and became more pronounced by week 4 (Figure 5H). Thus, chronic zolpidem treatment effectively suppressed Aβ accumulations. To validate the in vivo findings, we quantified amyloid plaque burden in postmortem brain sections following 4 weeks of daily treatment with either zolpidem or vehicle. Consistent with in vivo plaque burden data, zolpidem treatment was efficacious at reducing cortical plaque burden compared to that in vehicle‐treated controls in post‐mortem brain sections (Supplementary Figure 9).

### 3.8 Chronic zolpidem treatment decreased neuroinflammation and increased putative synaptic density in APP mice

To evaluate the safety of 30 mg/kg zolpidem, we assessed zolpidem's effect on[Fig alz71175-fig-0004], [Fig alz71175-fig-0005] neuroinflammation in the brain. We analyzed astrocytic and microglial responses following 4 weeks of daily treatment with either zolpidem or vehicle. Zolpidem‐treated mice showed reduced astrocytic and microglial reactivity in the cortex compared to that of vehicle‐treated controls (Supplementary Figure 10). Moreover, the putative cortical inhibitory synaptic density, characterized by the synaptic markers Bassoon, VGAT, and Gephyrin, was significantly increased following zolpidem treatment (Supplementary Figure 11). These results suggest that zolpidem did not induce detectable neuroinflammation or synaptotoxicity under the conditions tested. Instead, it reduced neuroinflammation and restored synaptic density.

### 3.9 Chronic zolpidem treatment reduced the percentage of neuronal processes with calcium overload in APP mice

Calcium homeostasis is necessary for proper neuronal function. Neuronal calcium homeostasis is impaired in APP mice. A subset of cortical neurons exhibits calcium overload, or significant elevations in basal calcium, due to amyloid β‐induced dysregulation, particularly within neurites, or neuronal processes of APP mice.^39^, ^40^ To evaluate whether chronic zolpidem treatment can restore neuronal calcium homeostasis, we expressed the ratiometric calcium indicator Yellow Cameleon 3.6 (YC3.6) in the cortex of APP mice. YC3.6 is a genetically encoded FRET‐based sensor containing YFP and CFP fluorophores. YC3.6 allows determination of real‐time intracellular calcium levels. Neurites were classified as overloaded when the YFP/CFP ratio exceeded 1.79, corresponding to intracellular calcium concentrations greater than 235 nM, or two standard deviations above the mean of neurites in nontransgenic controls.^27^

**FIGURE 6:**
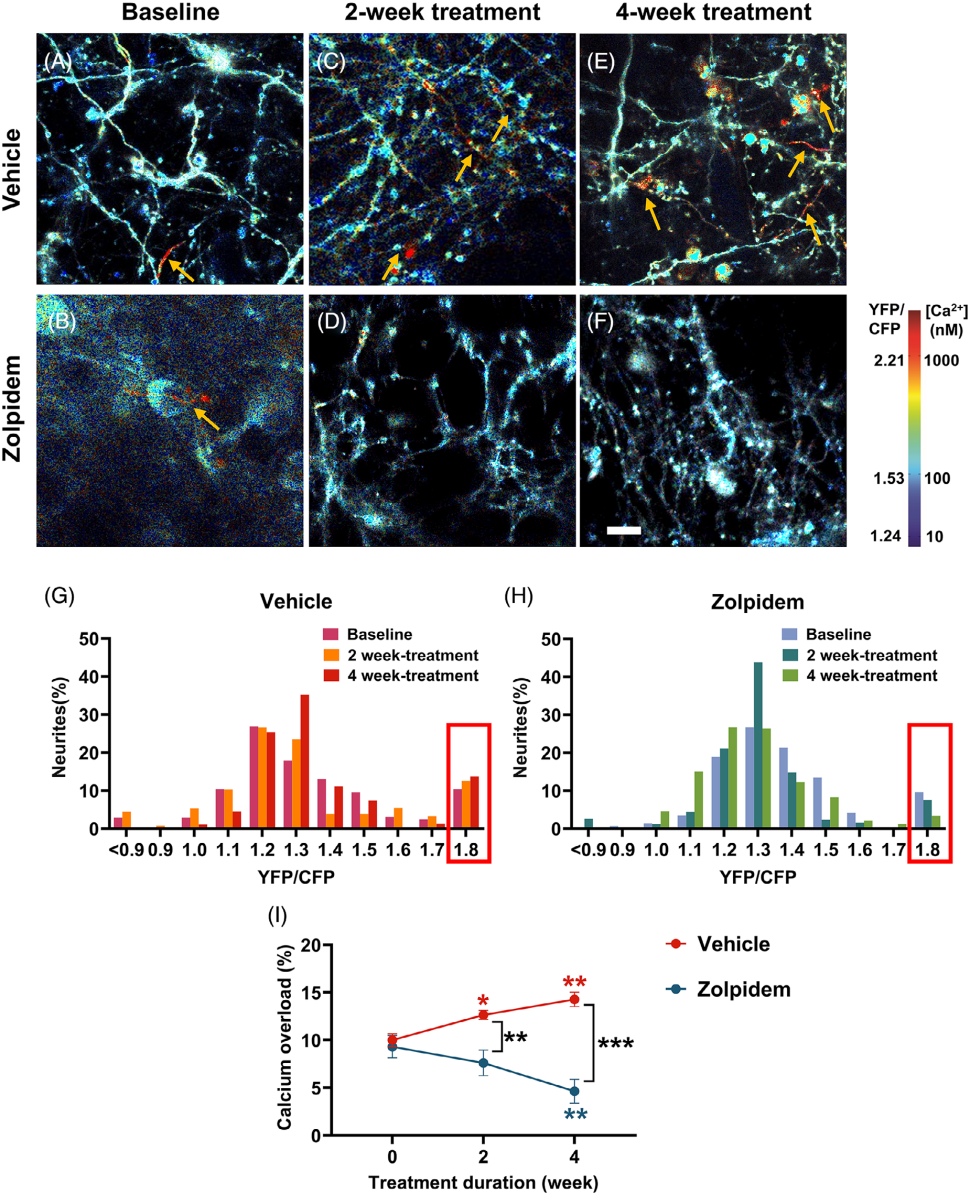
Zolpidem restored neuronal calcium homeostasis in APP mice. (A–F) Representative images of YC3.6‐labeled neurites showing calcium overload (red) in vehicle and zolpidem groups at baseline, 2 weeks, and 4 weeks. Images A–F were acquired at the same magnification. Scale bar, 50 µm. (G,H) Histograms of YFP/CFP ratios within neurites treated with vehicle (G) and zolpidem (H). (I) Quantification of calcium‐overloaded neurites (%) across groups. Mixed‐effects analysis followed by Tukey's multiple comparisons test. Blue* indicates significant differences compared to baseline within the zolpidem group. Red* indicates significant differences compared to baseline within the vehicle broup. Black* indicates significant group differences between zolpidem and vehicle treatments. Data are shown as mean ± SEM (*n* = 6–7 mice/group). *p < 0.05; **p < 0.01; ***p < 0.001. APP, amyloid precursor protein; YFP/CFP, yellow fluorescent protein/cyan fluorescent protein.

Representative images show numerous, red‐labeled neurites with calcium overload (yellow arrows) in vehicle‐treated mice at 2 and 4 weeks (Figure 6A,C,E). However, zolpidem‐treated mice showed a marked reduction in overloaded neurites over the same time period (Figure 6B,D,F). Histograms of YFP/CFP ratio distributions showed a progressive increase in the percentage of calcium‐overloaded neurites in the vehicle‐treated group over time (Figure 6G, red box). However, zolpidem‐treated mice exhibited a gradual reduction in the percentage of overloaded neurites over the treatment period (Figure 6H, red box). Quantitative analysis demonstrated that the percentage of neurites exhibiting calcium overload increased in vehicle‐treated mice from baseline to week 2 and increased further by week 4. However, zolpidem treatment significantly reduced the percentage of neurites with calcium overload by week 4, compared to baseline and vehicle controls (Figure 6I). These findings suggest that chronic zolpidem treatment restored calcium homeostasis and prevented calcium overload in neurites of APP mice, providing evidence for its potential neuroprotective effects beyond sleep regulation.

### 3.10 Chronic zolpidem treatment improved contextual recall in APP mice

**FIGURE 7:**
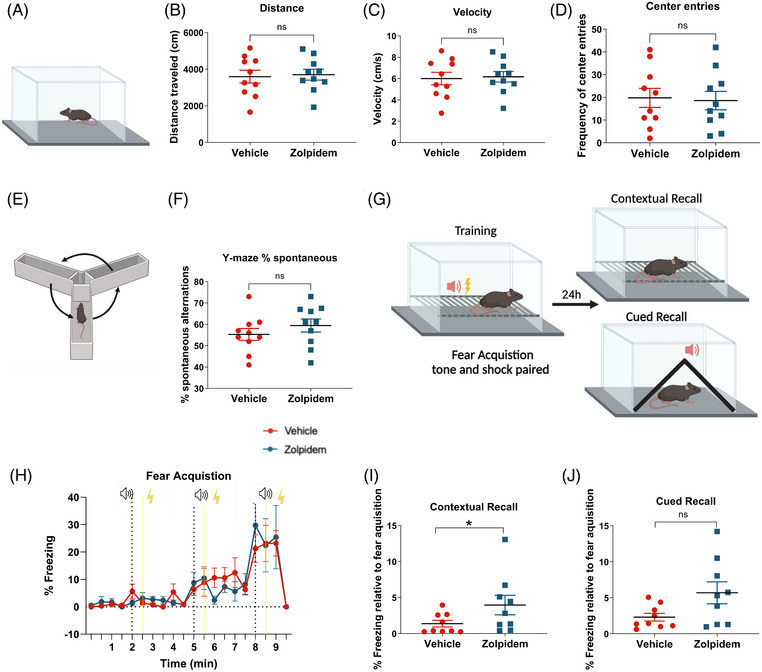
Zolpidem improved contextual memory without affecting locomotor activity or working memory. (A) Experimental setup for open field. (B–D) Distance travelled (B), velocity (C) and center entries (D) as a measure of open field performance across groups. (E) Experimental setup for Y‐maze test. (F) Y‐maze alternation rates across groups. (G) Contextual fear conditioning setup. (H–J) Fear acquisition (H), contextual recall (I) and cued recall (J) across groups. Unpaired two‐tailed t‐test. Data are shown as mean ± SEM (*n* = 9–10 mice/group). *p < 0.05.

To determine whether chronic zolpidem treatment alters cognitive function in APP mice, we conducted a series of behavioral assays, including open field, Y‐maze, and fear conditioning tests, following 4 weeks of daily treatment with either 30 mg/kg zolpidem or vehicle. Zolpidem‐ and vehicle‐treated mice exhibited comparable total distance traveled, average velocity, and frequency of center entries in the open field test (Figure 7A–D), indicating no significant differences in locomotor activity or anxiety‐like behaviors between groups. Similarly, no significant differences were observed in spontaneous alternations in the Y‐maze test (Figure 7E, F), suggesting that zolpidem treatment did not impact working memory in APP mice under these conditions.

We next assessed sleep‐dependent contextual recall using a classical fear conditioning paradigm (Figure 7G). During the acquisition phase, both groups exhibited increased freezing behavior over time (Figure 7H), indicating successful formation of fear memory. Then animals were placed back into their home cages and allowed to consolidate fear memory during sleep. Twenty‐four hours later, contextual fear recall was tested by reintroducing mice to the original training environment without any tone or shock. Zolpidem‐treated mice demonstrated significantly higher freezing levels during contextual recall compared to vehicle‐treated mice (Figure 7I), suggesting improved sleep‐dependent memory formation. In contrast, no significant differences were observed between groups during cued recall in a novel context (Figure 7J). These results suggest that chronic zolpidem administration selectively enhances sleep‐dependent memory formation in APP mice without affecting general locomotion or working memory performance.

## 4 DISCUSSION

The current work evaluated whether pharmacologically enhancing sleep‐dependent brain rhythms using FDA‐approved hypnotic zolpidem could restore cortical slow oscillation, improve NREM sleep duration, and reduce amyloid in APP mice. Acute zolpidem boosted slow oscillation power and increased sleep duration. Chronic administration reduced plaque burden, alleviated neuroinflammation, increased putative inhibitory synaptic density, normalized neuronal calcium homeostasis, and improved contextual recall without affecting locomotor or general cognitive functions. These results underscore the therapeutic potential of restoring sleep‐dependent brain rhythms to slow amyloidosis.

Early‐stage AD patients exhibit disruptions in NREM sleep.^9^, ^41^, ^42^, ^43^ Slow oscillation (0.5–1 Hz), consisting of alternating down‐states of neuronal silence and up‐states of synchronous firing,^13^, ^14^, ^15^ is essential for memory consolidation,^44^ synaptic homeostasis,^45^ and metabolic waste clearance.^46^ Impairments appear early in AD, often before substantial plaque deposition yet in the presence of oligomeric Aβ, suggesting a causal role in disease progression.^47^, ^48^, ^49^ Our published work demonstrated that slow oscillation is impaired in APP mice, with cortical desynchronization, neuronal calcium overload, and accelerated amyloid pathology. Optogenetic restoration of slow oscillation slowed amyloidosis, whereas further disruption worsened disease,^20^, ^21^ indicating slow oscillation is a viable therapeutic target.

Deficits in inhibitory tone also contribute to slow‐wave disruptions.^22^ Exogenous GABA restored slow oscillation in an AD mouse model, whereas pharmacological block of GABA_A_ and GABA_B_ receptors disrupted slow oscillation in healthy nontransgenic mice. Altered cortical inhibitory interneuron activity in APP mice further links interneuron dysfunction to impaired rhythms.^20^ We previously reported that rhythmic optogenetic stimulation of GABAergic interneurons at the endogenous frequency of slow oscillation, 0.6 Hz, restored slow waves and sleep in APP mice and slowed Alzheimer's progression,^22^ established a causal relationship between interneuron‐dependent impairments and Alzheimer's progression, its translational potential remained limited.

We therefore performed an unbiased computational screen of FDA‐approved compounds and revealed zolpidem's strong and stable binding to the GABA_A_ receptor at the BZD site, with structural dynamics similar to diazepam. Previously, Hanson and colleagues showed that zolpidem binds directly to the GABA_A_ receptor,^50^ supporting our approach and predicted models. MD simulations showed that zolpidem facilitated chloride ion transport across the membrane. High‐throughput docking and ML screening further prioritized zolpidem based on predicted binding affinity and potentiation of GABA signaling. These findings provided a mechanistic basis for testing zolpidem's potential to rescue disrupted network activity. Zolpidem, a GABA_A_ receptor modulator and proven hypnotic known to potentiate inhibition, promotes sleep onset and maintains sleep clinically.^51^, ^52^ Beyond its hypnotic effects, emerging evidence suggests that zolpidem has demonstrated beneficial effects in various neurological disorders, including stroke,^53^, ^54^ traumatic brain injury,^55^, ^56^ and Parkinson's disease.^57^, ^58^ Although zolpidem improves sleep quality in Alzheimer's patients, its impact on slow oscillation dynamics and AD pathology has not been thoroughly tested. Using in vivo VSD imaging, we identified 30 mg/kg as the most effective dose for subsequent chronic treatment experiments.

We next investigated whether zolpidem alleviated sleep impairments in APP mice, which displayed fragmented sleep architecture, increased wakefulness, and reduced NREM sleep, particularly during the light phase, mirroring abnormalities in Alzheimer's patients.^22^, ^59^, ^60^ EEG/EMG recordings showed that acute 30 mg/kg zolpidem significantly increased NREM sleep, specifically during the light phase, with effects dissipating by the following day. Zolpidem had minimal impact on sleep architecture during the dark phase, the animals' natural period of wakefulness, indicating that zolpidem selectively enhanced rest‐phase sleep without disrupting activity during the active phase.

Fourier transform analyses of EEG recordings during NREM sleep revealed dynamic changes induced by zolpidem. Consistent with our previous findings of cortical hyperexcitability characterized by reduced slow‐wave power and elevated higher‐frequency rhythms in APP mice, zolpidem significantly increased slow oscillation and delta power. Conversely, zolpidem suppressed higher‐frequency rhythms, transiently elevating beta activity. These changes indicate that zolpidem restores cortical excitatory‐inhibitory balance by promoting low‐frequency rhythms and modulating aberrant high‐frequency activities. These findings align with prior studies showing that zolpidem enhances slow‐wave power and suppresses high‐frequency EEG components during NREM sleep.^61^, ^62^, ^63^ However, not all studies report consistent outcomes, likely due to differences in disease models, dose, or analytics windows.^64^, ^65^

The temporal profile of zolpidem's effects differed between the VSD imaging and EEG recordings. In the VSD experiment, performed under light isoflurane anesthesia, slow‐oscillation power increased within 20 minutes after injection. In contrast, EEG recordings during natural sleep showed a delayed but more prolonged increase, emerging around 2 hours after treatment. These differences reflect the influence of anesthesia and methodological distinctions. Brief handling during the drug administration transiently increased arousal. Together, these complementary findings indicate that zolpidem enhances slow oscillatory activity, with the timing and duration shaped by the experimental context and brain state.

We assessed sleep and slow oscillation acutely because zolpidem alters sleep architecture and because sleep disruptions in APP mice are most pronounced at this stage,^22^ providing a window when intervention is most effective. Thus, chronic long‐term effects or zolpidem on sleep remain to be explored.

To determine whether the acute drug effects could translate into longer‐term benefits, we implemented a four‐weeks chronic dosing regimen. Longitudinal two‐photon imaging of amyloid plaques demonstrated that chronic zolpidem significantly suppressed amyloid deposition relative to vehicle‐treated controls. We used 7‐ to 9‐month‐old APP mice because by this age animals exhibited robust amyloid pathology and sleep impairments, allowing detection of treatment effects. Our prior studies demonstrated that interventions during early disease stages could effectively reverse sleep deficits.^22^ Our findings align with published studies indicating that impaired sleep exacerbates amyloid accumulation, whereas enhancing NREM sleep facilitates Aβ clearance.^42^, ^46^, ^66^ We also detected decreased neuroinflammation and increased putative synaptic density during the 4‐week treatment at the dose of 30 mg/kg zolpidem in APP mice.

In parallel, multiphoton calcium imaging revealed that chronic zolpidem treatment significantly reduced the proportion of neurites exhibiting calcium overload, a marker of synaptic dysfunction and neurotoxicity.^39^, ^40^ Reduced YFP/CFP ratios within neurites among zolpidem‐treated mice suggested improved intracellular calcium homeostasis, typically disrupted by elevated amyloid‐β levels and cortical hyperexcitability.^40^ The normalization of neuronal calcium levels was evident as early as 2 weeks into treatment and became more pronounced by week 4, indicating progressive, cumulative therapeutic effects.

Finally, to determine whether these neurophysiological improvements translated into functional gains, we conducted a battery of behavioral assays. Zolpidem‐treated APP mice displayed intact locomotor function and healthy working memory, as indicated by open field and Y‐maze, and exhibited enhanced contextual fear, suggesting that improved sleep and cortical network synchronization enhanced contextual recall.

Parhizkar and colleagues^67^ recently reported that Lemborexant improved tau‐associated sleep–wake impairments and brain atrophy, whereas zolpidem did not confer neuroprotection. Their work examined later AD stages using ∼9‐month‐old PS19 tau and human apolipoprotein E4 (APOE4) knock‐in mice with evident neuronal loss. In contrast, we used 6‐ to 7‐month‐old APP mice, which model amyloidosis without tau pathology and neuronal loss. Cortical circuits in our APP mice were hyperactive, whereas those in tau mice were hypoactive. Therefore, zolpidem restored circuit function by reducing circuit hyperexcitability in young APP mice but failed to rescue circuit dysfunction in tau mice due to pronounced circuit hypoactivity.

Although previous studies have reported potential adverse effects of zolpidem treatment, including cognitive impairment and increased risk of falls in older adults, these outcomes appear to be dose‐dependent and population‐specific,^68^, ^69^, ^70^ and are often associated with long‐term high‐dose exposure. Zolpidem is routinely prescribed to elderly populations and has demonstrated a favorable safety profile in short‐term intervention studies.^69^ A recent clinical trial found that zolpidem decreased wakefulness and increased sleep duration in Alzheimer's patients, with mild to no adverse events reported.^71^ Other clinical and preclinical studies have similarly reported beneficial effects of chronic zolpidem administration.^53^, ^72^ Intermittent low‐dose administration at early AD stages, when cortical circuits are hyperactive due to reduced inhibitory tone, may maximize benefit while minimizing sedation risk. Collectively, these observations highlight the importance of optimizing therapeutic strategies to maximize clinical benefit while minimizing potential risks. In addition, previous studies have tested zolpidem at doses up to 60 mg/ml^73^ demonstrating sedative effects without evidence of functional neurotoxicity. When comparing animal studies with human data, it is important to recognize the limitations of direct dose equivalence. Thus, the emphasis should be on identifying qualitative effects and defining relative safety margins. A comprehensive analysis of these aspects lies beyond the scope of the present study. Zolpidem did not induce detectable synaptic toxicity under the tested conditions. More extensive toxicity profiling remains an important avenue for future research.

Our initial goal was to validate the computational pipeline and establish proof‐of‐principle by focusing on Zolpidem. Evaluating additional compounds is the next step in the future. These studies will allow us to determine how broadly this computational analysis can be applied. Because APP mice do not fully replicate the diverse pathological features of AD, it will be important to validate findings across multiple AD models to ensure translational relevance. Furthermore, accumulating evidence indicates that the efficacy of sleep‐targeting drugs can differ substantially between males and females.^67^, ^74^ Although our study did not detect sex‐dependent differences during sleep recordings, the limited sample size may have constrained our ability to identify such effects. Future research should systematically investigate how sex influences the efficacy of sleep‐targeting drugs.

## CONFLICT OF INTEREST STATEMENT

The authors declare no conflicts of interest. Author disclosures are available in the Supporting Information.

## CONSENT STATEMENT

No human data were derived; therefore, consent was not necessary.

## Supporting information



Supporting Information

Supporting Information
