# Supplementary material for: Zolpidem restores sleep and decreases amyloid in a mouse model
Source: Alzheimers Dement. 2026 Mar 11;22(3):e71175. doi: 10.1002/alz.71175 (PMC12976976; doi:10.1002/alz.71175)
Supplement: Supplementary file 1 — Supporting Information [file ALZ-22-e71175-s001.pdf]

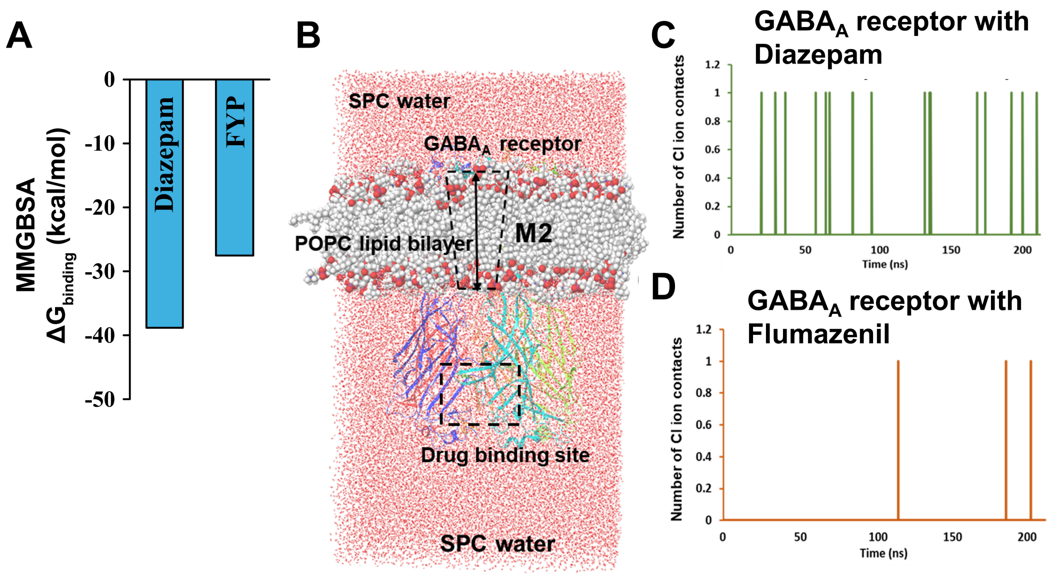

Supplementary Figure 1: (A) Predicted Gibbs binding free energies for diazepam and flumazenil (FYP). (B) Three-dimensional representation of the Simple Point Charge (SPC) water simulation system, illustrating the GABA$_A$ receptor embedded in a 1-palmitoyl-2-oleoyl-sn-glycero-3-phosphocholine (POPC) lipid bilayer and bound to either diazepam or flumazenil. (C) Number of chloride ions (Cl-) traversing the M2 transmembrane pore of the GABA$_A$ receptor bound to diazepam. (D) Number of chloride ions traversing the M2 pore of the receptor bound to flumazenil, calculated from 200 ns simulation trajectory frames.

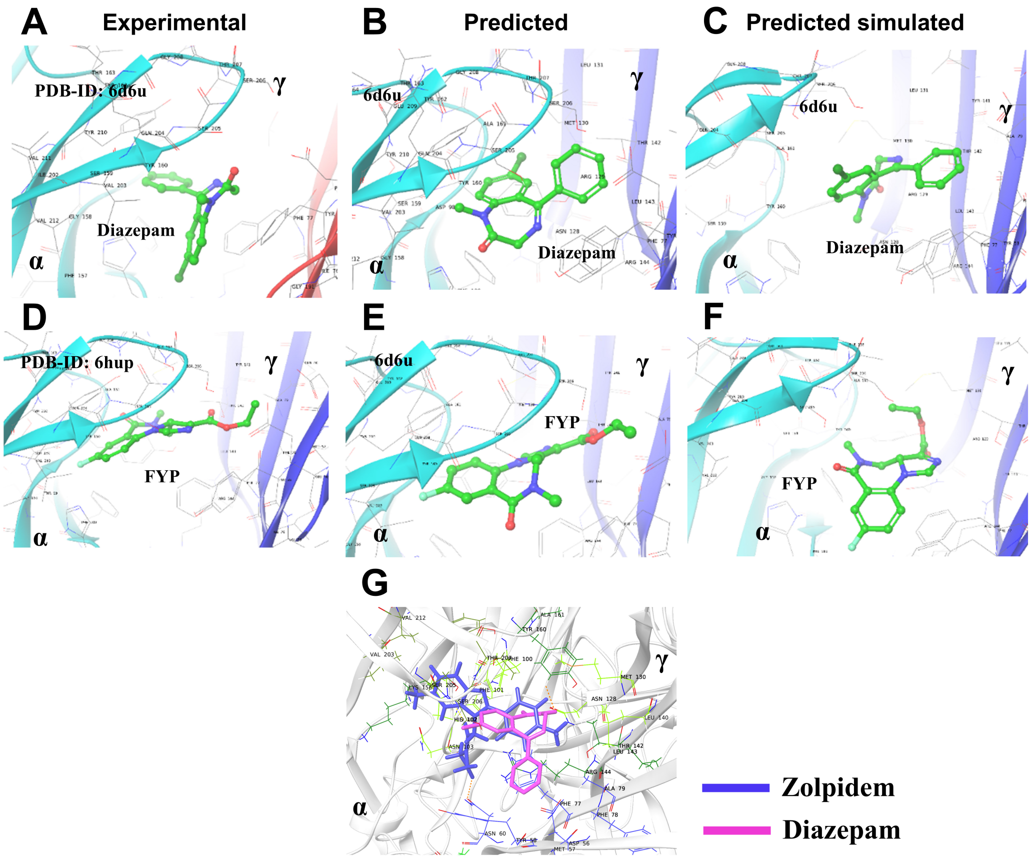

Supplementary Figure 2. Protein-ligand docking analysis of known ligand interactions to GABA$_A$ Receptor. (A) Representation of diazepam binding modes and interacting amino acids inferred from experimental structure (6hup). (B) Predicted interactions of diazepam using protein-ligand docking method. (C) Predicted interactions of diazepam after a short simulation of retrieved GABA$_A$ receptor structure. (D) Protein-ligand interaction analysis profiler depicting interaction mode and interacting amino acids with flumazenil, inferred from experimental structure (6d6u). (E) Predicted binding mode for flumazenil, depicting the binding region and interacting with amino acids. (F) Predicted interactions of flumazenil after a short simulation of retrieved GABA$_A$ receptor structure (6d6u). (G) Snapshot of predicted binding pose for zolpidem superimposed with diazepam binding pose with GABA$_A$ receptor structure.

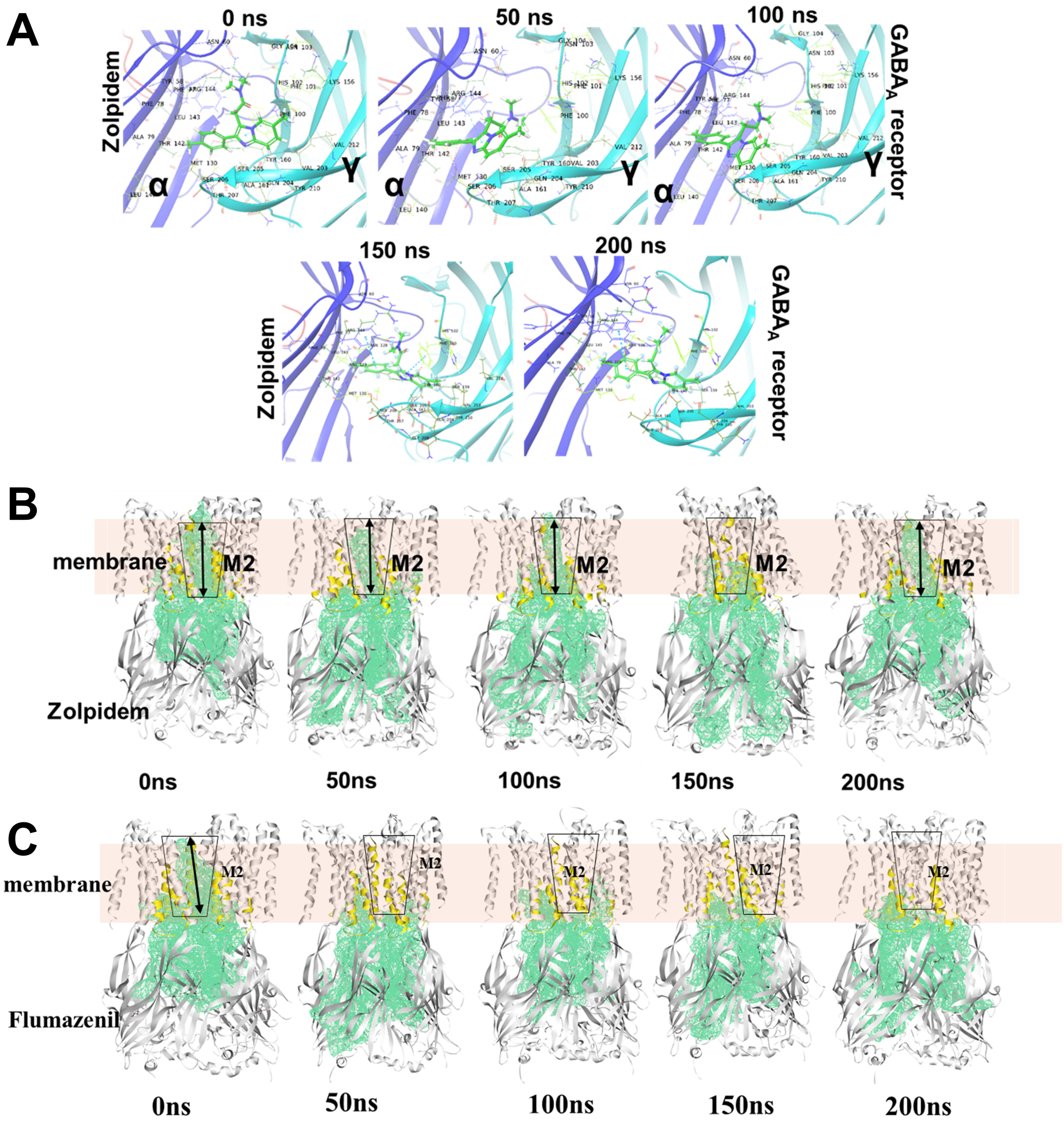

Supplementary Figure 3. Atomistic Molecular Dynamic Simulation of the $GABA_A$ Receptor Bound to Zolpidem. (A) Representative snapshots from trajectory frames captured at various time points during the 200 ns simulation of the $GABA_A$ receptor in complex with zolpidem. (B) Three-dimensional structural representations of the $GABA_A$ receptor bound to zolpidem at selected time intervals, analyzed for conformational changes in the membrane-embedded cavity (membrane pore) induced by ligand binding. (C) Three-dimensional structural snapshots of the $GABA_A$

receptor bound to flumazenil, taken at different time points, depicting conformational changes within the membrane-embedded cavity (membrane pore).

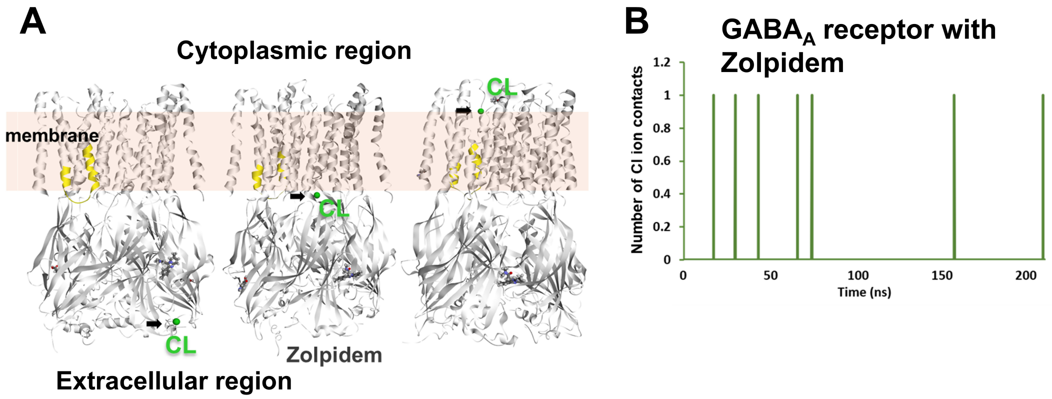

Supplementary Figure 4. Trajectory Analysis of the GABA$_A$ Receptor–Zolpidem Complex from Molecular Dynamics Simulations. (A) Three-dimensional representations of the GABA$_A$ receptor bound to zolpidem, illustrating chloride ion transport across the membrane at selected time points. (B) Total number of chloride ion contacts with the M2 transmembrane region of the GABA$_A$ receptor during the simulation, indicating ion conduction behavior in the presence of zolpidem.

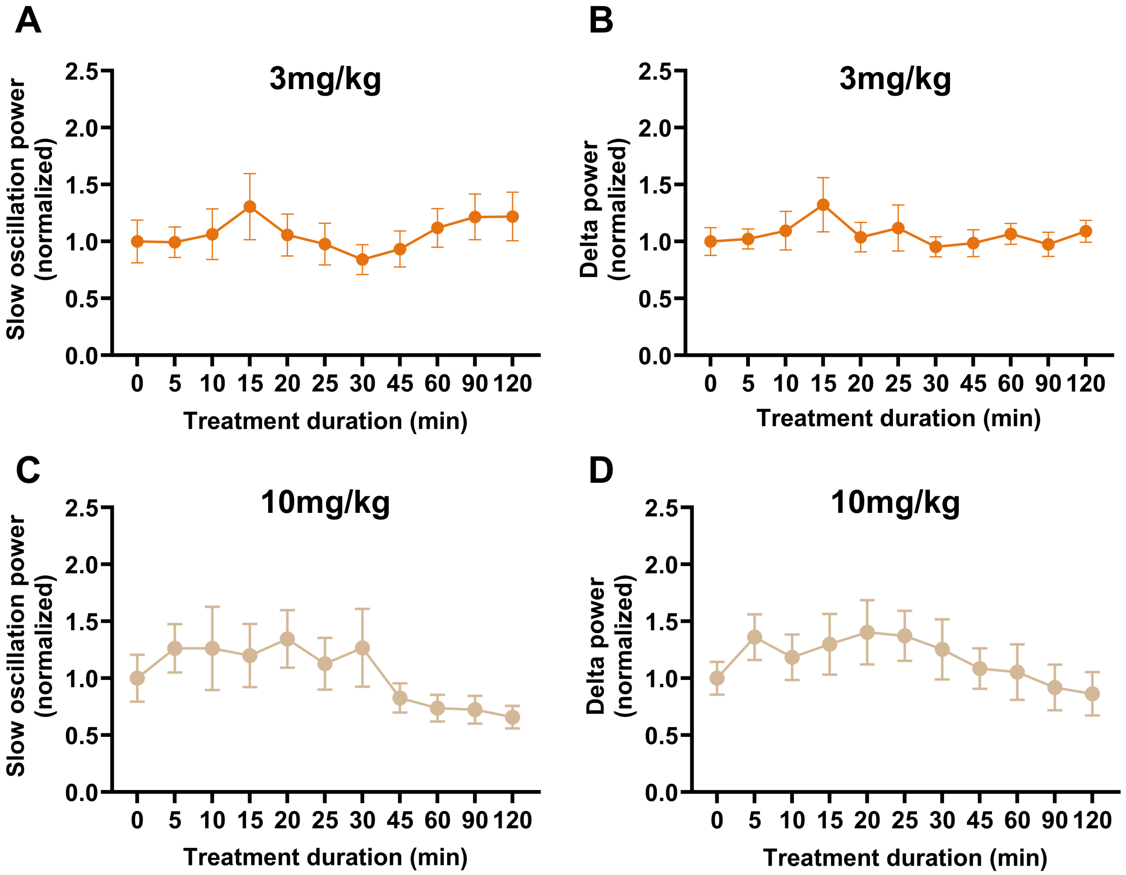

Supplementary Figure 5. Time course of slow oscillation and delta power changes following 3 and 10 mg/kg zolpidem administration in APP/PS1 mice. (A–B) 3 mg/kg zolpidem did not significantly alter slow oscillation power (A) or delta power (B) over 120 min post-injection. (C–D) 10 mg/kg zolpidem did not significantly increase slow oscillation power (C) and delta power (D). Friedman test followed by Dunn's multiple. Data is shown as mean ± SEM (*n* = 5-6 mice/group); power was normalized to baseline (0 min). Lack of stars indicates lack of significance.

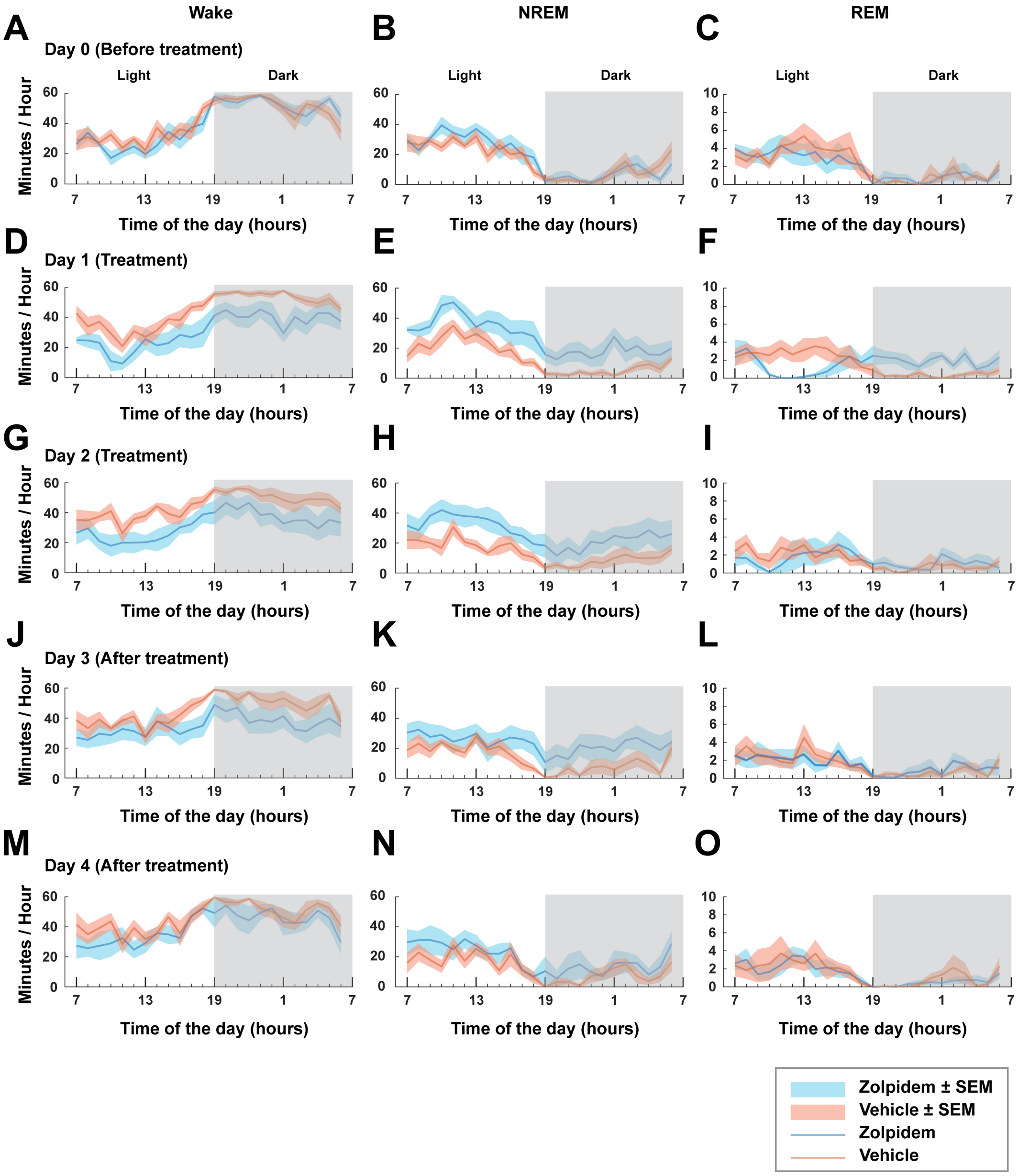

Supplementary Figure 6. 24-hour plots of wake, NREM, and REM sleep architecture across light and dark phases. Time courses of wake (A, D, G, J, M), NREM (B, E, H, K, N) and REM (C, F, I, L, O) sleep states in APP/PS1 mice treated with vehicle (red) or zolpidem (blue). Sleep and wake states were monitored before treatment, (Day 0; A–C), during treatment, (Day 1; D–F, Day 2; G–I), and after treatment (Day 3; J–L, Day 4; M–O). Data is represented as mean ± SEM (*n* = 6–7 mice per group).

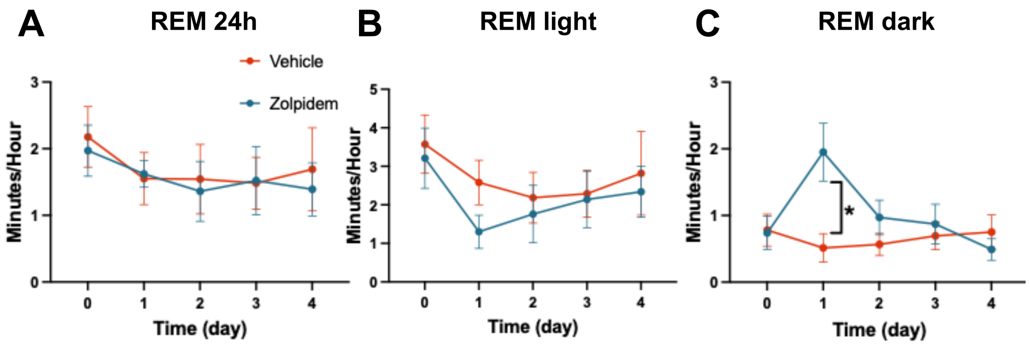

Supplementary Figure 7. REM sleep durations across five days in APP/PS1 mice treated with vehicle or zolpidem. (A) No significant difference in total 24-hour REM sleep between groups. (B) No significant difference in light phase of REM sleep between groups. (C) REM sleep during the dark phase increased in the zolpidem group on Day 1 compared to the vehicle. Mixed-effects analysis followed by Tukey's multiple comparisons test. * indicates significant group differences between zolpidem and vehicle treatments. Data is represented as mean ± SEM (n = 6-7 mice/group). *$p < 0.05$. Lack of stars indicates lack of significance.

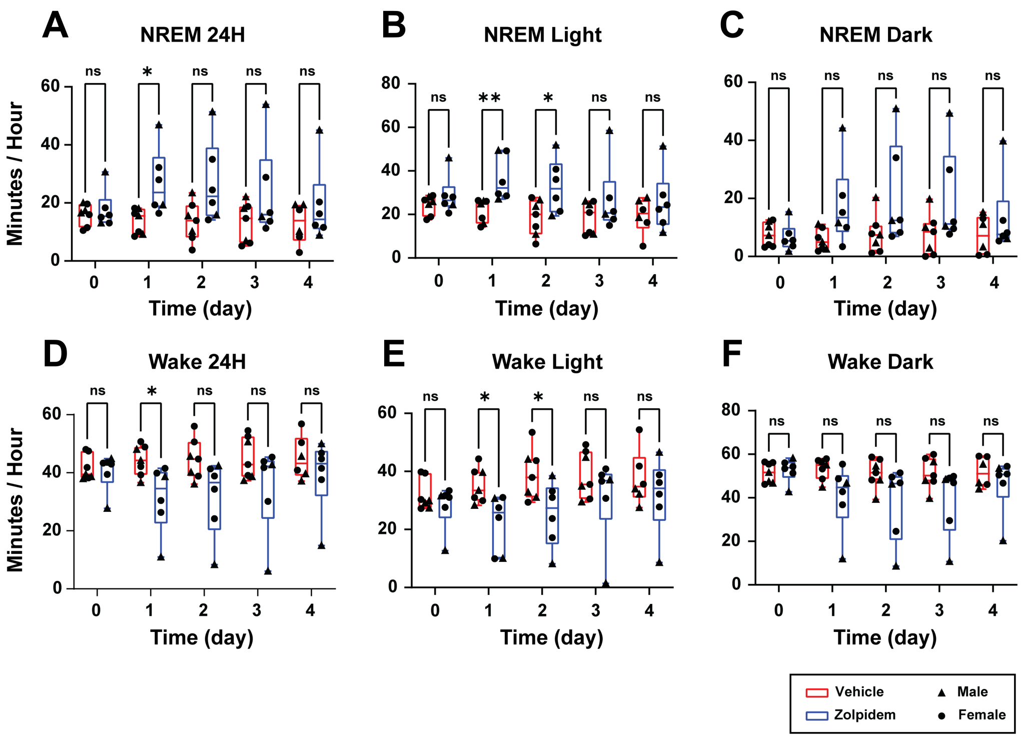

Supplementary Figure 8. NREM sleep and wake duration distributions across male and female mice after vehicle or zolpidem treatment. (A–C) Individual data and quantification of NREM sleep durations across 24 hours (A), light phase (B), and dark phase (C). (D–F) Individual data and quantification of wakefulness durations across 24 hours (D), light phase (E), and dark phase (F). Sleep and wake states were monitored before treatment (Day 0), during treatment (Day 1 and Day 2), and after treatment (Day 3 and Day 4). Data is represented as mean ± SEM (n = 6–7 mice per group). Circles represent males, triangles represent females. n.s., not significant; *p < 0.05, **p< 0.01.

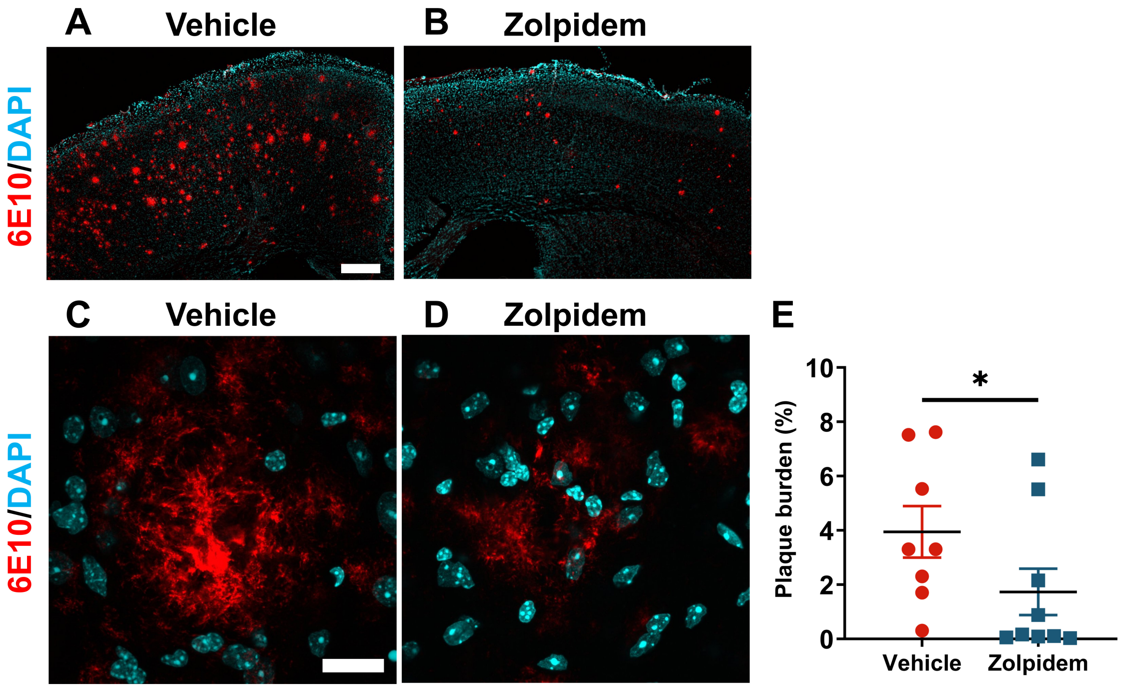

Supplementary Figure 9. Zolpidem treatment reduced cortical amyloid plaque burden following 4 weeks of daily administration. (A–D) Immunofluorescent staining of amyloid plaques using the 6E10 antibody. (E) Quantification of cortical plaque burden, calculated as the percentage of cortical area occupied by 6E10 positive area. Statistical analysis was performed using Student's t-test ($p = 0.103$). Scale bars: 400 µm (A) and 20 µm (C). Data is represented as mean ± SEM ($n = 8$–9 mice per group).

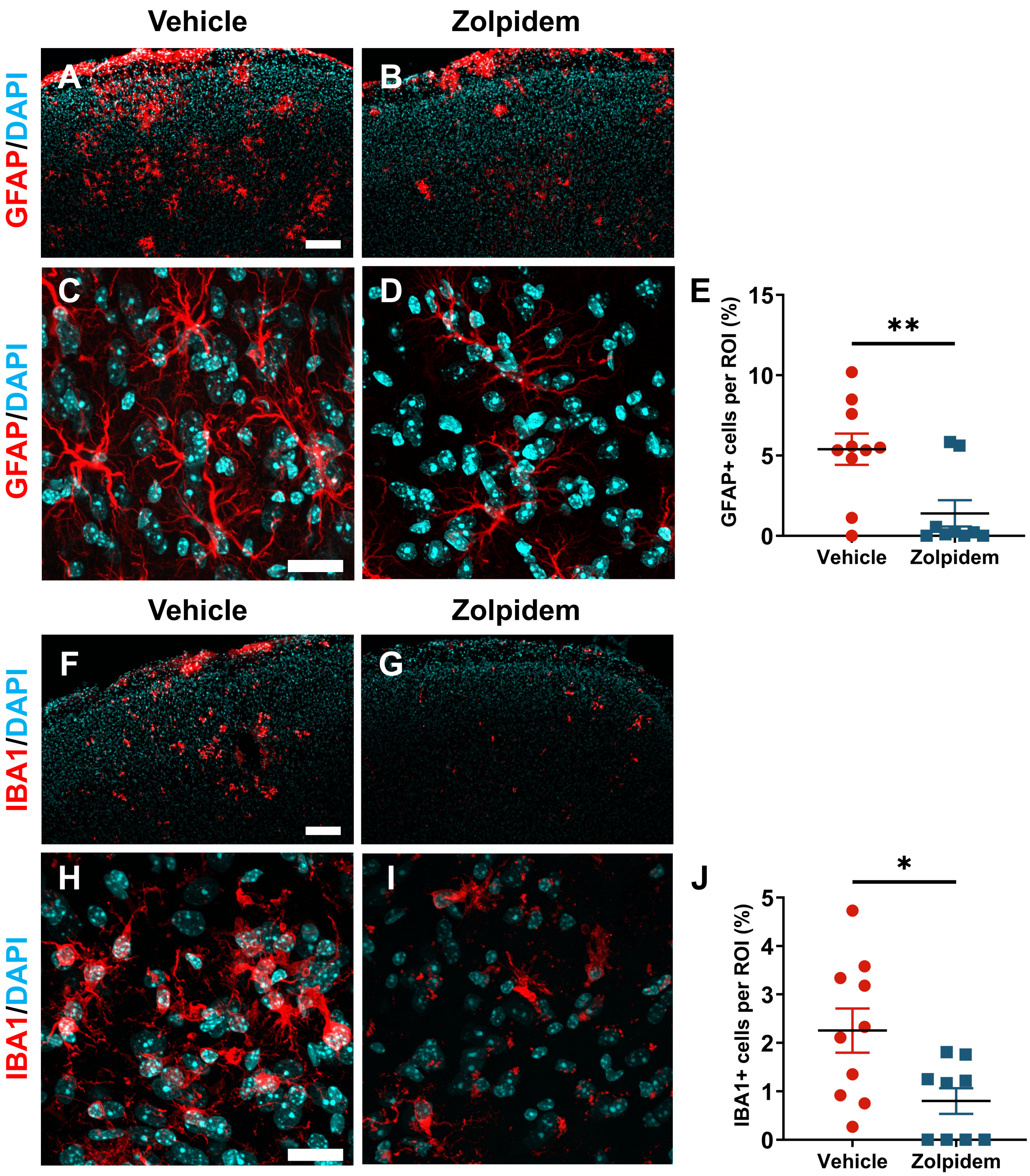

Supplementary Figure 10. Reduced astrocyte and microglial activation in the cortex following 4 weeks of daily zolpidem treatment. (A–D) Immunofluorescent staining for reactive astrocytic marker GFAP. (E) Quantification of GFAP^{+} cells per ROI, calculated as the percentage of cortical area occupied by GFAP positive area. Statistical analysis was performed using Student's t-test (p = 0.0065). (F–I) Immunofluorescent staining for reactive microglial marker Iba1. (J)

Quantification of $Iba1^+$ cells per ROI, calculated as the percentage of cortical area occupied by Iba-1 positive area. Statistical analysis was performed using Student's t-test ($p = 0.0159$). Scale bars: 400 µm (A, F) and 20 µm (C, H). Data is represented as mean ± SEM ($n = 9$–10 mice per group). *$p < 0.05$, **$p < 0.01$.

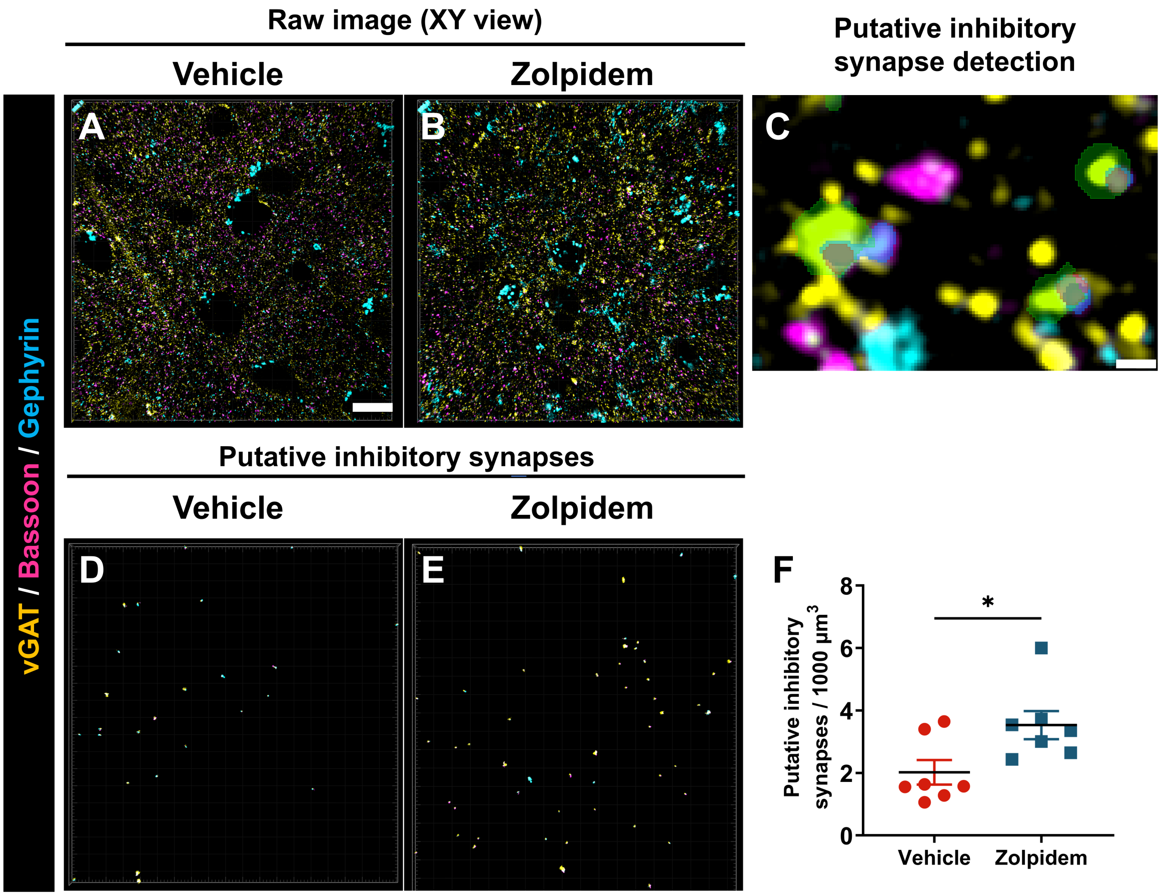

Supplementary Figure 11. Zolpidem treatment restored cortical putative inhibitory synaptic density following 4 weeks of daily administration. (A–B) SIM images showing labeling of synaptic markers VGAT, Bassoon, and Gephyrin in the cortex. (C) Detection of putative inhibitory synapses using the Blob Finder operation and object filtering. Object filters were applied as follows: Bassoon objects must be located within VGAT objects or exhibit >50% overlap; Gephyrin objects must be attached to or overlapping Bassoon objects. (D–E) Distribution of putative inhibitory synapses. (F) Quantification of synaptic density (per volume). *n* = 7–8 mice per group. Each data point represents the mean of 3–4 ROIs per mouse. Data is presented as mean ± SEM. Brown–Forsythe and Student's t-tests were applied (*p* = 0.027). Scale bars: 10 µm (A) and 400 nm (C).

99
